# Perceived support from AI chatbots and loneliness among higher vocational college students: a short-term three-wave study of social interaction anxiety and perceived social support

**DOI:** 10.3389/fpsyg.2026.1928350

**Published:** 2026-09-15

**Authors:** Jiangnan Hu

**Affiliations:** Yingtan Vocational and Technical College, Yingtan, China

**Keywords:** artificial intelligence, higher vocational college students, loneliness, perceived social support, perceived support from AI chatbots, short-term three-wave design, social interaction anxiety

## Abstract

Generative artificial intelligence and AI companion tools are increasingly entering higher vocational college students' learning, emotion regulation, and everyday social communication contexts. This study defines perceived support from AI chatbots as students' subjective experience of receiving support while using AI chat tools, rather than objective social support from real interpersonal sources. A short-term three-wave design was used. Students were recruited through class WeChat groups from four higher vocational colleges in Jiangxi and Guangdong provinces, China, during the spring semester of the 2025–2026 academic year. The intervals between T1, T2, and T3 were approximately 8 weeks. The valid T1 sample included 1,500 participants, and the core three-wave matched sample included 1,200 participants. The revised observed-composite cross-lagged panel model showed that T2 perceived support from AI chatbots negatively predicted T3 loneliness. At the same time, T1 and T2 perceived support from AI chatbots positively predicted social interaction anxiety at the following wave, and social interaction anxiety further predicted loneliness at the following wave. The longitudinal mediation analysis indicated that T1 perceived support from AI chatbots indirectly predicted T3 loneliness through T2 social interaction anxiety. T1 perceived social support moderated the associations of T2 perceived support from AI chatbots with T3 loneliness and T3 social interaction anxiety: under high perceived social support, perceived support from AI chatbots was more likely to be associated with lower subsequent loneliness; under low perceived social support, this compensatory association was not significant. These findings suggest that the psychological meaning of support from AI chatbots is conditional. AI literacy and mental health education in colleges should attend to how AI-based support can be connected with real social support networks.

## 1 Introduction

Loneliness is not merely a lack of objective social contact. Rather, it reflects a distressing discrepancy between the social relationships individuals desire and the social relationships they perceive in reality (Hawkley and Cacioppo, 2010). Because loneliness is closely related to emotional distress, social withdrawal, depressive symptoms, and functional impairment, it has been recognized as an important public health issue (Cacioppo and Cacioppo, 2018). This study focuses on higher vocational college students, but this focus does not imply that AI-related support issues are limited to college settings. Instead, higher vocational college students constitute an important subgroup of emerging adults and higher education students. They are simultaneously facing adaptation away from home, peer-network reconstruction, academic tasks, career preparation, and highly digitalized communication contexts. Thus, this sample provides a suitable context for examining the short-term longitudinal association between perceived support from AI chatbots and loneliness in a group where AI use is rapidly becoming routine and psychological adaptation demands are concentrated.

Digital interaction may provide lonely individuals with opportunities to reconnect with others, but it may also deepen social isolation when it displaces offline relationships (Nowland et al., 2018). With the rapid spread of generative AI and AI companion tools, the relationship between AI chatbots, AI companions, and loneliness has become an important topic in digital mental health research (Montag et al., 2026; Shelmerdine and Nour, 2025; Taebnia, 2025). Unlike ordinary social media or search tools, AI systems can provide immediate, non-judgmental, and continuously available responses, making them easy for users to perceive as companion-like and supportive (Jacobs, 2024). However, such support is not equivalent to real interpersonal support. AI chatbots cannot assume reciprocal relationships, shared activities, or long-term obligations in the same way as family members, friends, or peers. Therefore, this study uses the term perceived support from AI chatbots to emphasize a subjective support experience in an AI context rather than interpersonal social support in the traditional sense.

However, perceived support from AI chatbots may also be accompanied by risks related to social interaction anxiety. The social compensation, social enhancement, and displacement hypotheses in digital communication research suggest that the psychological consequences of online interaction depend on whether it expands existing social relationships or crowds out offline interaction opportunities (Kraut et al., 2002; McKenna et al., 2002; Valkenburg and Peter, 2007). This logic can be cautiously extended to AI-support contexts. When students use AI responses for emotional organization, problem clarification, and preparation for real help-seeking, AI support may have compensatory significance. When students are more inclined to obtain low-pressure responses through AI interaction while reducing real social practice or offline help-seeking, AI support may be associated with later social discomfort. It should be emphasized that this study did not directly measure reductions in offline interaction or AI substitution for real relationships. Therefore, this article uses the terms “social-interaction-anxiety-related risk association” or “substitution-risk interpretation” to describe this result, rather than treating it as direct evidence of actual substitution behavior.

This issue is particularly important in higher education contexts. Students are increasingly using AI systems as sources of emotional, academic, and social support (Nazir, 2025). Research on AI-based mental health solutions for international students suggests that social support conditions may influence the effectiveness of AI tools (Amin et al., 2025). At the same time, reviews of AI in mental health, clinical evidence reviews, and conceptual frameworks emphasize that the accessibility advantages of AI tools must be evaluated together with privacy, safety, governance, clinical boundary, and referral risks (Ali et al., 2025; Balan and Gumpel, 2025; Boit and Patil, 2025; Estrada-Araoz, 2026; Rezaei et al., 2026; Umasugi and Fitriasari, 2026). Case-based and risk-focused discussions further suggest that interactions with AI chatbots may induce or reinforce specific psychological risks, which requires careful boundaries of use and crisis referral mechanisms (Hudon and Stip, 2025). Accordingly, AI support should be understood as a psychosocial exposure, not merely as a technical service.

This study treats social interaction anxiety as a candidate psychological pathway linking perceived support from AI chatbots to loneliness, rather than assuming in advance that AI support necessarily causes social anxiety. Social interaction anxiety refers to fear, discomfort, and avoidance experienced in interpersonal situations, and the Social Interaction Anxiety Scale provides a mature measure of this construct (Mattick and Clarke, 1998). A three-wave longitudinal study showed that social anxiety can transmit other psychological risks to later loneliness (Akkus and Peker, 2026). A three-wave cross-lagged study of stress and loneliness also showed that loneliness among college students has temporal stability and may be affected by contextual stress (Lu et al., 2025b). Existing digital behavior research is not fully consistent regarding the direction between social anxiety and loneliness; some studies treat loneliness as a mediator between social anxiety and problematic digital behavior (Zhou and Feng, 2025; Fang et al., 2025). In the CLPM, this study therefore retained theoretically relevant adjacent-wave cross-lagged and reverse paths and interpreted the results as short-term longitudinal predictive associations.

Perceived social support in real life may determine whether perceived support from AI chatbots is closer to a supplementary resource or an isolated substitute-like experience. Perceived social support refers to the extent to which individuals believe that they can obtain help from family, friends, and significant others (Zimet et al., 1988). Digital social interaction is more likely to reduce loneliness only when it promotes real social connection and support. If online interaction is disconnected from real relationship networks, its protective role may be limited (Nowland et al., 2018). Because perceived social support was measured only at T1, MSPSS was treated in this study as a baseline time-invariant resource rather than a dynamic moderator. This design allows the boundary role of initial real-life support conditions to be tested, but it cannot demonstrate how real-life support changes together with AI use during the semester.

Generative AI use has become embedded in everyday higher education. Students' adoption of generative AI is jointly shaped by social norms, institutional contexts, and perceived usefulness (Ursavas et al., 2025). Research on generative AI in higher education indicates that teachers and students perceive both supportive functions and substitution-related concerns associated with AI (Chea and Deng, 2026; Haroud and Saqri, 2025). Empirical research further shows that students' use of generative AI is no longer merely an occasional trial but is becoming part of routine learning (Carmi, 2025; Sousa and Cardoso, 2025). Related studies also suggest that generative AI may alter teaching processes, motivational appraisals, and student wellbeing (Jukiewicz, 2025; Kinskofer and Tulis, 2025). Therefore, AI literacy frameworks emphasize that students need not only access to AI tools but also the ability to judge when AI should and should not be used (Hackl et al., 2026; Tarhini et al., 2026).

Based on this literature, the present study focuses on three progressive questions. First, after controlling for prior loneliness, social interaction anxiety, and depressive symptoms, does perceived support from AI chatbots predict subsequent changes in loneliness? Second, is perceived support from AI chatbots linked to the development of loneliness through subsequent social interaction anxiety? Third, does perceived social support in real life function as a baseline protective condition that moderates the associations between perceived support from AI chatbots and later psychological outcomes? Compared with existing digital communication research, the theoretical contribution of this article is to extend the social compensation/social enhancement perspective, the displacement hypothesis, and the social support buffering model to the context of generative AI support, proposing that perceived support from AI chatbots may simultaneously show compensatory associations and social-anxiety-related risk associations.

## 2 Theoretical background and hypotheses

### 2.1 Perceived support from AI chatbots and loneliness: compensatory and risk associations

The social compensation perspective suggests that individuals with fewer real-life social resources or obstructed offline interactions may obtain relational resources through online interaction. The social enhancement perspective suggests that online resources are more easily transformed into relational benefits by individuals who already have higher social resources (Kraut et al., 2002; McKenna et al., 2002). In contrast, the displacement hypothesis emphasizes that if digital interaction replaces offline contact and the acquisition of real support, it may weaken rather than enhance social connection (Valkenburg and Peter, 2007; Nowland et al., 2018). Because support from AI chatbots does not come from real interpersonal sources, this study does not simply equate it with traditional online social support. Instead, it examines how this perceived support in an AI context predicts subsequent loneliness and social interaction anxiety in the short term.

According to the social compensation perspective, perceived support from AI chatbots may be associated with lower subsequent loneliness. AI systems can provide immediate responses, opportunities for emotional expression, informational suggestions, and companion-like experiences, and therefore may temporarily compensate for support deficits for some students (De Freitas et al., 2024). A qualitative study of older adults showed that AI chatbots can respond to loneliness through a sense of conversational presence (Jiao et al., 2026). Longitudinal evidence on AI chatbot companionship also suggests that, in some contexts, AI companionship may be associated with reductions in loneliness and psychological distress (Suroyo et al., 2026). Research on autistic users similarly treats chatbot companionship as an accessible form of loneliness support (Hollis et al., 2026). However, the effect of AI companionship on loneliness may be heterogeneous across users and may depend on user type, cultural context, and real-life social support conditions (Liu et al., 2025; Qian et al., 2026; Uguz et al., 2026). Together, these findings support a compensatory pathway of AI support, while also suggesting that this compensatory function should not be understood as an unconditional protective effect.

According to the displacement hypothesis and substitution-risk interpretation, perceived support from AI chatbots may also be related to later discomfort in real social situations. Young people may be more willing to seek support from AI chatbots on sensitive issues because AI is more controllable and involves lower evaluation pressure (Monselise et al., 2026). Research on generative AI for personal psychological support suggests that repeated linguistic interactions and dialogue patterns may allow users to form stable support routines (Shih, 2026). This study did not directly measure opportunities for social-skill practice, offline interaction frequency, or actual substitution behavior. Therefore, this pathway is defined as a “social-interaction-anxiety-related risk association,” rather than causal evidence that AI use replaces real relationships.

Therefore, throughout this article, the term “social-interaction-anxiety-related risk association” refers only to the statistical association between perceived support from AI chatbots and higher social interaction anxiety at the following wave, together with the subsequent predictive association between social interaction anxiety and loneliness. It should not be interpreted as evidence that AI support reduced offline interaction, replaced real relationships, or caused students to avoid real social situations. This wording keeps the theoretical interpretation aligned with the variables actually measured in this study.

**H1:** After controlling for prior loneliness, social interaction anxiety, depressive symptoms, and demographic variables, perceived support from AI chatbots will negatively predict loneliness at the following wave, indicating a compensatory longitudinal predictive association.
**H2:** After controlling for prior social interaction anxiety, loneliness, depressive symptoms, and demographic variables, perceived support from AI chatbots will positively predict social interaction anxiety at the following wave, indicating a social-anxiety-related risk longitudinal predictive association.

### 2.2 Social interaction anxiety as a longitudinal mediating pathway

Research on problematic AI use provides a theoretical basis for the substitution pathway. Problematic ChatGPT use can be understood in terms of its manifestations, etiologies, and assessment needs (Liao et al., 2026). Scale development research similarly suggests that when AI assistance shifts toward problematic dependence, the negative side of AI-human collaboration may emerge (Maral et al., 2025). Research on large language model addiction among university students further links excessive LLM involvement with mental health problems (Elshaer and Azazz, 2025). These studies indicate the need to examine whether perceived support from AI chatbots temporally predicts subsequent social interaction anxiety.

Social anxiety and loneliness are closely related among students, but the direction of this association may be recursive. Loneliness mediates the association between social anxiety and smartphone addiction among college students (Zhou and Feng, 2025), and problematic mobile social network use may also be linked to social anxiety through loneliness and self-concept clarity (Fang et al., 2025). Research on developmental trajectories of loneliness, smartphone addiction, and social anxiety among college students further indicates that these psychological and digital behavior variables may change together over time (Chen et al., 2025). Longitudinal research has also shown cross-temporal associations between loneliness and problematic social media use (Wu et al., 2024), and physical activity, social anxiety, loneliness, and mobile phone addiction may form a chain process (Zhu et al., 2025). These findings suggest that loneliness, social anxiety, and digital behaviors may mutually reinforce each other rather than operate in a single direction. Because this study focuses on the development of loneliness, social interaction anxiety was treated as a candidate mediating pathway between perceived support from AI chatbots and later loneliness, while theoretically relevant reverse paths were also reported in the CLPM to avoid a one-directional interpretation.

**H3:** Social interaction anxiety will positively predict loneliness at the following wave.
**H4:** Social interaction anxiety will mediate the longitudinal association between perceived support from AI chatbots and subsequent loneliness. This hypothesis tests an indirect predictive association in temporal order rather than a strict within-person causal mechanism.

### 2.3 Perceived social support as a boundary condition

The social support buffering model argues that social support can reduce the impact of stress or risk exposure on mental health by providing emotional reassurance, problem-solving resources, and a sense of belonging (Cohen and Wills, 1985). In the context of AI support, students with higher real-life perceived social support may be more likely to use AI responses for help-seeking preparation, emotional organization, and relational communication, allowing perceived support from AI chatbots to supplement real support networks. Students with lower real-life perceived social support may lack the conditions needed to translate AI responses into actual help-seeking and relational action. Because MSPSS was measured only at T1, this study examines baseline real-life support conditions rather than the dynamic interaction between real-life support and AI support.

Perceived social support is expected to buffer the social-interaction-anxiety-related risk associated with AI support. Online social support is closely related to digital psychological processes among college students (Jiang et al., 2023). Real-life relational resources may also influence digital-use risks, as shown in longitudinal research on family functioning, problematic social media use, and loneliness (Lu et al., 2025a). Social support, social anxiety, loneliness, and depressive symptoms may also form a chain psychological process (Tang and Lu, 2026). A study comparing ChatGPT with validated questionnaires further suggests that loneliness and online social support among college students can be empirically assessed (Gu et al., 2025). These studies support treating perceived social support as a protective boundary condition rather than merely as a routine control variable.

**H5:** Perceived social support will negatively predict subsequent social interaction anxiety and loneliness.
**H6:** T1 perceived social support will moderate the associations between perceived support from AI chatbots and subsequent outcomes. Specifically, when real-life perceived social support is higher, the negative association between perceived support from AI chatbots and subsequent loneliness will be stronger, and the positive association with subsequent social interaction anxiety will be weaker.

### 2.4 The present study

This study constructs and tests a short-term three-wave model to examine how perceived support from AI chatbots is associated with loneliness among higher vocational college students. Drawing on the social compensation/social enhancement perspective, the displacement hypothesis, and the social support buffering model, this study proposes that perceived support from AI chatbots may have dual psychological meanings. On the one hand, supportive responses from AI may provide compensatory resources for lonely students through emotional reassurance, informational guidance, and conversational companionship. On the other hand, continuous low-pressure support experiences from AI may also be associated with increased discomfort in real interpersonal interaction, which may further predict later loneliness risk.

This study advances existing research in three respects. First, prior work has mainly focused on social media use, smartphone use, or general online social support, whereas this study focuses on AI-mediated supportive experiences and explicitly defines them as perceived support in an AI context. Second, this study does not assume that AI support is simply beneficial or harmful. Instead, it tests a dual-pathway model that distinguishes a compensatory pathway, in which AI support may predict lower loneliness, from a social-interaction-anxiety-related risk association, in which AI support may predict higher loneliness through social interaction anxiety. Third, this study includes T1 real-life perceived social support as a boundary condition to examine whether real-life support resources shape the short-term longitudinal meaning of AI support.

Using three-wave data, this study examines whether perceived support from AI chatbots directly predicts subsequent loneliness, whether social interaction anxiety functions as a longitudinal mediator between perceived support from AI chatbots and loneliness, and whether T1 real-life perceived social support moderates these associations. In this model, perceived support from AI chatbots is positioned as a dual-pathway predictor of loneliness: one pathway is compensatory, whereby perceived support from AI chatbots predicts lower subsequent loneliness; the other is a social-interaction-anxiety-related risk association, whereby perceived support from AI chatbots predicts higher subsequent social interaction anxiety, and social interaction anxiety further predicts higher subsequent loneliness. The conceptual model is shown in Figure 1.

**Figure 1:**
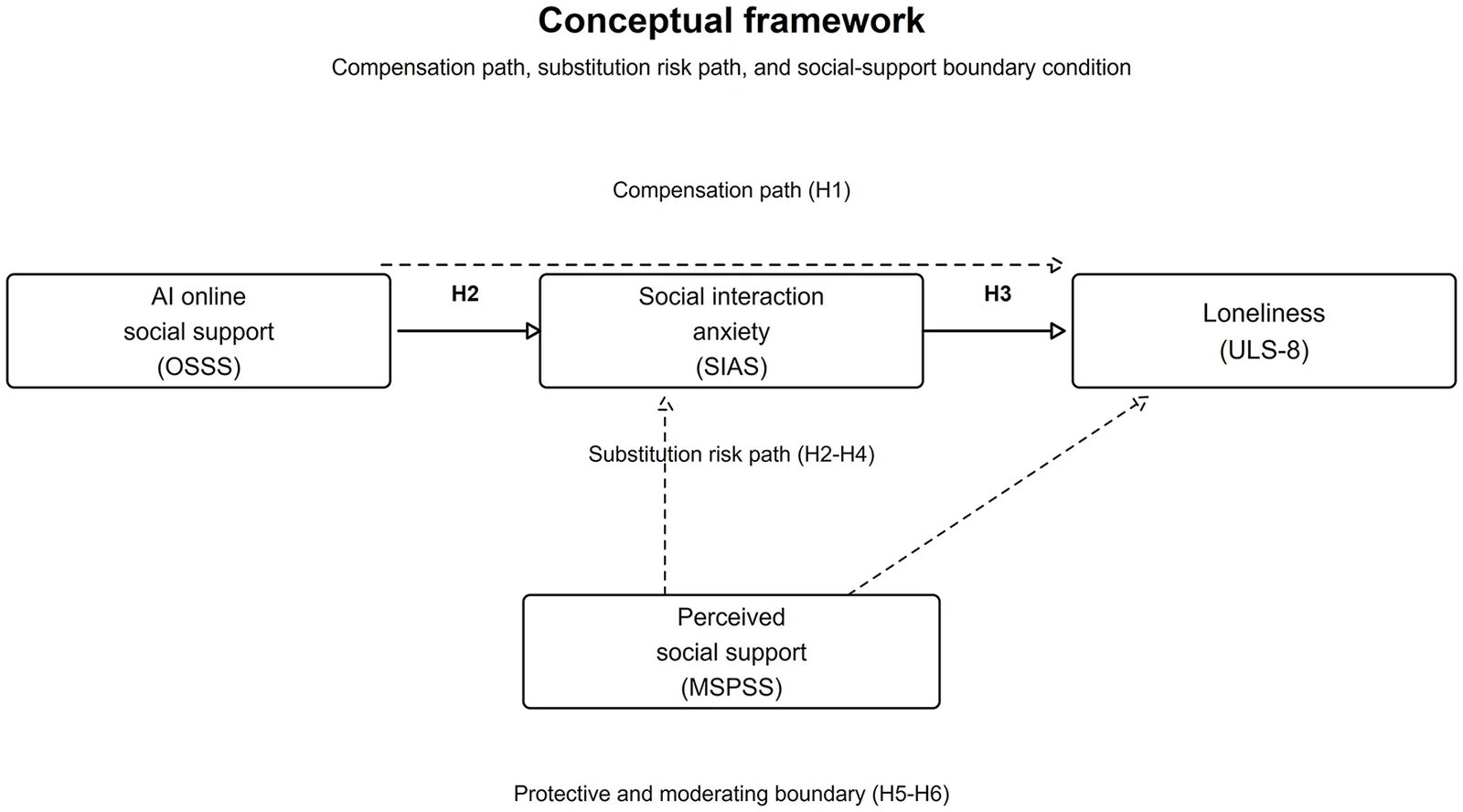
Conceptual model. The model distinguishes the compensatory pathway between perceived support from AI chatbots and subsequent loneliness, the risk association via social interaction anxiety, and the protective and moderating roles of real-life perceived social support. OSSS, SIAS, ULS-8, and PHQ-9 were repeatedly measured across three waves; MSPSS was measured at T1.

## 3 Materials and methods

### 3.1 Participants and procedure

This short-term three-wave longitudinal panel study was conducted during the spring semester of the 2025–2026 academic year in four higher vocational colleges in Jiangxi and Guangdong provinces, China. Cluster sampling was used, with intact classes as the recruitment units, and stratification was based on major category. Online survey links were distributed by course instructors through class WeChat groups. The original questionnaire system and exported dataset did not retain class identifiers that could accurately assign participants to specific classes. Therefore, the number of participating classes at each institution and class-level sample sizes could not be reconstructed, and class-level ICCs and design effects could not be estimated. Because only four institutions were included, institution-level ICCs, multilevel models, or cluster-robust standard errors would also lack stability. Thus, subsequent models were estimated at the individual level, and this limitation is further discussed in the Section 5.4. Accordingly, although this study reports available individual-level longitudinal predictive associations, the standard errors, *p* values, and significance levels should be interpreted cautiously because potential class clustering could not be statistically adjusted.

To further clarify the implications of cluster recruitment for statistical inference, it should be noted that intact-class recruitment and questionnaire distribution through class WeChat groups may have made students from the same class more similar in teacher reminders, peer norms, AI-use contexts, and access to mental-health resources. If such within-class similarity existed and was positive, conventional individual-level models may have underestimated standard errors and produced overly optimistic p values and significance levels. Because the original data did not retain anonymous class identifiers, this study could not use cluster-robust standard errors or multilevel models to statistically adjust for this non-independence. Therefore, the present interpretation emphasizes path direction, effect size, consistency across model specifications, and sensitivity analyses rather than relying on a single significance test as the sole criterion.

Data were collected at 3 time points: T1 during weeks 2–3 of the semester, T2 during weeks 10–12, and T3 during weeks 16–18. The interval between adjacent waves was approximately 8 weeks, and the full follow-up period covered one semester. Thus, this study should be understood as a short-term three-wave design suitable for testing short-term longitudinal predictive associations after controlling for prior levels.

The inclusion criterion was self-reported use of at least one generative AI chat tool (e.g., ChatGPT, Doubao, ERNIE Bot, Kimi, Tongyi Qianwen, or DeepSeek) within the 3 months before T1. A screening item at the beginning of the T1 questionnaire asked respondents to select the AI chat tools they had used in the past 3 months. Those who selected “none of the above” were excluded.

To match participants across waves while reducing the risk of identity exposure, all participants were asked to generate the same self-identification code at each time point (format: last four digits of mobile phone number + birth month; for example, a participant whose phone number ended in 0523 and who was born in July entered “052307”). This code was de-identified/pseudonymous matching information rather than fully anonymous information. It was used only for cross-wave data matching and not for identity recognition. According to the ethics approval and data management plan, the original matching codes were accessible only to the principal investigator and designated data administrator, stored in encrypted files, and kept separately from questionnaire response data. Course instructors, counselors, and institutional administrators had no access to individual matching codes or individual responses. The matching codes will be retained for no longer than 6 months after completion of T3 data collection. After three-wave matching, duplicate-record checking, and data cleaning are completed, files containing the original matching codes will be deleted according to the data management procedure. The analysis dataset retains only random study IDs and de-identified variables. Any external sharing will be limited to de-identified data and analysis syntax.

At T1, 1,639 students accessed the survey link. After excluding 97 respondents who did not pass the screening item and 42 with clearly invalid response patterns (straight-line responding or completion time < 3 min), the valid T1 sample included 1,500 students. At T2, 1,324 responses were received, and 1,200 participants provided valid matched data at both T1 and T2. At T3, 1,413 responses were received, and the final core complete three-wave matched sample included 1,200 participants (three-wave retention rate = 80.0%). To address data consistency concerns, this article uniformly reports descriptive statistics and main model results based on the complete matched sample for the three-wave core variables (*N* = 1,200). Wave-specific access numbers, valid sample sizes, matched sample sizes, and exclusion reasons are presented in the participant-flow information.

No financial compensation, course credit, lottery, certificate, or other material incentive was provided. Across the three waves, voluntary participation notices and gentle reminders in the same format were posted in class WeChat groups. The reminders emphasized that participation or non-participation would not affect course grades, teacher evaluations, or school services. Participants could withdraw from the study at any wave without providing a reason.

In the final analytic sample (*n* = 1,200), 60.2% were female, and the mean age was 19.43 years (SD = 1.28, range = 17–24). The grade distribution was 38.3% first-year students, 35.7% second-year students, and 26.0% third-year students. Major categories were education (22.5%), engineering and technology (28.3%), business administration (25.7%), humanities and arts (15.8%), and other majors (7.7%). Most participants (79.2%) lived on campus. The average daily use of AI chat tools was 38.5 min (SD = 42.7), with 21.3% reporting less than 15 min, 35.2% reporting 15–30 min, 25.1% reporting 30–60 min, 13.2% reporting 1–2 h, and 5.2% reporting more than 2 h.

The study protocol was approved by the Ethics Review Committee of Yingtan Vocational and Technical College (approval number: YTZYJSXY20260931). All participants provided electronic informed consent before entering the formal questionnaire. The study followed an ethics-approved de-identified, low-risk electronic survey procedure and did not collect directly identifying information such as names or student ID numbers. Cross-wave matching codes were used only for data matching and were stored separately in encrypted files by the principal investigator and designated data administrator with strictly restricted access. Teachers, counselors, and course administrators could not view individual matching codes or individual responses. Matching codes will be retained for no longer than 6 months after completion of T3 data collection. After matching, duplicate-record checking, and data cleaning are completed, files containing the original matching codes will be deleted according to the data management procedure. At the end of the questionnaire, all participants were provided with contact information for the psychological counseling center and crisis support resources. Participants reporting higher-risk responses on PHQ-9 item 9 additionally received psychological crisis help-seeking prompts and referral information.

### 3.2 Measures

All questionnaires were administered in simplified Chinese. Existing measures with validated Chinese versions were used directly. The core variable, perceived support from AI chatbots, was assessed using a contextually adapted version of an existing validated scale. For all Likert-type scales, higher scores represented higher levels of the construct, and reverse-scored items were recoded before total scores were computed.

#### 3.2.1 Perceived support from AI chatbots

Perceived support from AI chatbots was measured using an AI-context-adapted Chinese short form of the Online Social Support Scale (OSSS-CS) (Zhou and Cheng, 2022). The OSSS-CS is a 20-item Chinese scale refined through factor analysis on the basis of the original Online Social Support Scale (Nick et al., 2018). It assesses four dimensions of online social support, with five items per dimension: esteem/emotional support, social companionship, informational support, and instrumental support. It should be noted that the original scale referred to support from “people online,” whereas the support source in this study was AI chat tools. Therefore, this adaptation should not be viewed as a fully equivalent minor wording change. This construct was defined as students' subjective perception of supportive experiences while using AI chat tools, rather than social support from real interpersonal sources in the traditional sense.

In this study, the contextual referent of each item was changed from “online” or “people online” to “AI chat tools” or “on AI chat tools.” For example, the original item “People online show concern for me” was adapted as “When using AI chat tools, I feel cared for.” For items involving shared activities or interpersonal interaction, the construct definition explicitly states that the adapted scale does not measure traditional interpersonal social support. Instead, it captures subjective supportive perceptions in an AI context. Therefore, interpretation of the results is limited to perceived supportive experiences during the use of AI chat tools. This scale is better understood as “perceived support in an AI context,” which may include perceived responsiveness, emotional companionship, informational help, instrumental assistance, and conversational satisfaction. Full item-level validation and discriminant validity from AI dependence, anthropomorphism, and AI-use intensity remain to be further established. Future research should also examine the discriminant validity of this construct in relation to AI dependence, anthropomorphism, emotional attachment to AI, perceived responsiveness, and general AI-use intensity, to avoid simply equating perceived support in an AI context with human social support.

From the perspective of construct validity, the evidence provided in this study mainly supports the content appropriateness, internal consistency, basic structure, and feasibility of cross-wave total-score comparison for the adapted scale in the present sample. These forms of evidence do not mean that its full construct network has been sufficiently established. Perceived support from AI chatbots may overlap with several adjacent constructs. For example, AI dependence reflects difficulty controlling use or psychological attachment; anthropomorphism reflects the attribution of human-like qualities to AI; emotional attachment to AI reflects relational investment; perceived responsiveness reflects the extent to which users believe AI understands and responds to their needs; and general AI-use intensity reflects use frequency or duration. Therefore, future research should measure these adjacent constructs simultaneously and use discriminant-validity tests, competing measurement models, multitrait-multimethod designs, or latent-variable models to further determine whether perceived support from AI chatbots has independent theoretical and measurement meaning.

Participants rated each item on a 5-point Likert scale (1 = never, 2 = rarely, 3 = sometimes, 4 = often, 5 = always). The instruction was: “Please respond based on your actual experiences of using AI chat tools during the past 2 months.” The same 20-item OSSS was repeatedly administered at T1, T2, and T3, and total scores were calculated separately for each wave. Total scores ranged from 20 to 100, with higher scores indicating greater perceived social support from AI chat tools. Four dimension scores, each based on five items (range = 5–25), could also be calculated.

The adapted scale was reviewed by three educational psychologists and pilot-tested in a small sample of 200 higher vocational college students to examine item clarity and appropriateness for the AI context. Pilot feedback indicated that participants could understand the item meanings after the support source was changed from “people online” to “AI chat tools,” but this adaptation still requires further full item-level validity evidence in future research. Confirmatory factor analysis (CFA) using T1 data supported the four-factor structure: χ^2^ (164) = 482.36, *p* < 0.001; CFI = 0.943; TLI = 0.934; RMSEA = 0.040 [90% CI (0.036, 0.044)]; SRMR = 0.042. Because the OSSS was repeatedly administered at T1, T2, and T3, this study further tested longitudinal measurement invariance. Given the large number of parameters in a full 20-item, three-wave, four-factor longitudinal item model, the main text reports multi-group CFA results based on the four theoretical dimension scores. Changes in fit indices for configural, weak, and strong invariance were within commonly used criteria, supporting the basic appropriateness of comparing total scores across waves. It should be emphasized that the very high CFI/TLI values in Supplementary Table S1 may be related to the use of subscale indicators, low model degrees of freedom, and indicator aggregation, and cannot replace a full item-level longitudinal latent measurement model. Total scores showed good internal consistency across the three waves (Cronbach's α: T1 = 0.94, T2 = 0.93, T3 = 0.94).

#### 3.2.2 Loneliness

Loneliness was assessed using the short form of the UCLA Loneliness Scale (ULS-8) (Hays and DiMatteo, 1987). The ULS-8 includes eight items assessing subjective loneliness and the frequency of social isolation (e.g., “I lack companionship” and “I feel left out”). Items are rated on a 4-point Likert scale (1 = never, 2 = rarely, 3 = sometimes, 4 = always). Three positively worded items are reverse scored (e.g., “I am an outgoing person”). Total scores range from 8 to 32, with higher scores indicating greater loneliness. The ULS-8 was derived from the full UCLA Loneliness Scale (Russell, 1996), is highly correlated with the 20-item full version (*r* > 0.90), and has received psychometric support in Chinese adolescent or undergraduate samples (Xu et al., 2018; Wu and Yao, 2008). Cronbach's α values in this study were 0.85 at T1,0.84 at T2, and.87 at T3.

#### 3.2.3 Social interaction anxiety

Social interaction anxiety was measured using the Social Interaction Anxiety Scale (SIAS) (Mattick and Clarke, 1998). The SIAS is a 20-item self-report measure assessing anxiety experienced in social interaction situations (e.g., “I have difficulty making eye contact with others” and “I feel tense mixing with people I do not know well”). Participants rated items on a 5-point Likert scale (0 = not at all characteristic of me, 4 = extremely characteristic of me). One positively worded item (“I find it easy to make friends of my own age”) was reverse scored. Total scores range from 0 to 80. The SIAS and related short-form tools have shown supportive psychometric evidence in Chinese college samples (Ouyang et al., 2020). Cronbach's α values in this study were 0.90 at T1, 0.92 at T2, and 0.92 at T3.

#### 3.2.4 Perceived social support

Real-life perceived social support was measured using the Multidimensional Scale of Perceived Social Support (MSPSS) (Zimet et al., 1988). The MSPSS is a 12-item instrument assessing perceived support availability from three sources: family, friends, and significant others. Items are rated on a 7-point Likert scale (1 = very strongly disagree, 7 = very strongly agree). Total scores range from 12 to 84, with higher scores indicating higher real-life perceived social support. MSPSS was measured only at T1 and was included as a baseline time-invariant resource variable. The total score rather than the three subscale scores was used because this study focused on overall social support resources as a buffering factor and needed to control model complexity. However, different support sources may have different roles. Future studies should repeatedly measure support from family, friends, and significant others and test their dynamic moderating effects separately. Cronbach's α in this study was 0.92.

#### 3.2.5 Depressive symptoms

Depressive symptoms were assessed using the Patient Health Questionnaire-9 (PHQ-9) (Kroenke et al., 2001). The PHQ-9 includes nine items corresponding to the symptom criteria for major depressive disorder and uses a 4-point frequency scale (0 = not at all, 3 = nearly every day). Total scores range from 0 to 27. The PHQ-9 has shown satisfactory diagnostic validity among Chinese college students (Zhang et al., 2013). Depressive symptoms were included in the main model as a time-varying psychological distress variable measured at all three waves, to reduce confounding effects of general emotional distress on pathways involving loneliness and social interaction anxiety. Because PHQ-9 scores may also be related to perceived support from AI chatbots and loneliness, the results should be interpreted as longitudinal predictive associations adjusted for general psychological distress, rather than as unadjusted overall associations. Cronbach's α values were 0.88 at T1, 0.89 at T2, and 0.89 at T3.

#### 3.2.6 Demographic and AI-use variables

Participants reported gender, age, grade, major category (science/engineering, humanities, education, business administration, arts, or other), residence status (living on campus vs. commuting), AI chat tool use duration, and primary purpose of use (academic help, emotional conversation, problem solving, entertainment, writing/translation, information retrieval, or other). Major category was treated as a nominal variable and entered into formal models using dummy coding or effect coding rather than as a continuous ordinal variable. AI-use duration and primary purpose were used mainly for sample description. Because AI-use purpose involved multiple categories, some subgroups were unevenly distributed, and a full multi-group CLPM would substantially increase model complexity, this study did not conduct multi-group cross-lagged models by AI-use purpose. The second-round supplementary analyses focused on covariate coefficients that could be stably estimated, including demographic variables, residence status, depressive symptoms, and baseline real-life perceived social support, and tested the robustness of focal paths under different covariate adjustment specifications.

### 3.3 Data analysis

Analyses were conducted using R and Mplus 7. First, descriptive statistics, internal consistency coefficients, and Pearson correlations were calculated. Table 1 reports descriptive statistics based on the complete matched sample for the three-wave core variables (*N* = 1,200), and the structural models were estimated using the same complete matched sample. OSSS, social interaction anxiety, loneliness, and depressive symptoms were treated as repeated measures across three waves. Perceived social support (MSPSS) was measured only at T1 and entered the model as a baseline time-invariant resource variable. To examine cross-wave measurement comparability, longitudinal measurement invariance tests were conducted for OSSS, SIAS, and ULS-8 by sequentially evaluating configural, weak, and strong invariance. OSSS used four theoretical dimension scores as indicators, whereas SIAS and ULS-8 used fixed item parcels as indicators. A three-wave observed-composite cross-lagged panel model (CLPM) was then estimated, including autoregressive paths, adjacent-wave cross-lagged paths, within-wave residual correlations, and theoretically relevant reverse paths. The model controlled for gender, age, grade, major category, residence status, depressive symptoms, and T1 real-life perceived social support. AI-use duration and primary purpose were used for sample description. Given category distributions, subgroup sample sizes, and model complexity, they were not included in the second-round supplementary sensitivity models. Categorical covariates were dummy coded or effect coded. Because the initial CLPM showed inadequate fit, the revised model added second-order autoregressive paths from T1 to T3 for the same constructs in addition to adjacent-wave autoregressive paths, to control for stability components spanning two measurement intervals. Longitudinal mediation was tested using bias-corrected bootstrap confidence intervals. The interpretation followed recommendations on temporal ordering and prior-level control in longitudinal mediation models (Cole and Maxwell, 2003). In addition, because traditional CLPMs cannot strictly distinguish stable between-person differences from within-person change, path coefficients were interpreted as longitudinal predictive associations after controlling for prior levels rather than as strict within-person causal effects (Hamaker et al., 2015). The moderation analysis mainly tested whether T1 real-life perceived social support moderated the associations of T2 OSSS with T3 loneliness and T3 social interaction anxiety. All interaction terms were constructed from standardized variables. Because the original data did not retain class identifiers, class-level ICCs, design effects, cluster-robust standard errors, or multilevel models were not estimated. This means that the model results reflect individual-level longitudinal predictive associations.

**Table 1:** Descriptive statistics.

Variable	*N*	*M*	SD	Minimum	Maximum	Skewness	Kurtosis
T1 perceived support from AI chatbots	1,200	59.06	13.40	20	100	−0.05	−0.10
T2 perceived support from AI chatbots	1,200	59.41	12.92	20	94	−0.10	−0.13
T3 perceived support from AI chatbots	1,200	59.21	12.69	20	98	−0.05	−0.06
T1 social interaction anxiety	1,200	41.13	12.96	0	80	0.03	0.06
T2 social interaction anxiety	1200	33.96	11.83	0	80	0.05	−0.18
T3 social interaction anxiety	1,200	34.62	13.02	0	80	0.24	0.07
T1 loneliness	1,200	20.85	4.18	8	32	0.01	−0.36
T2 loneliness	1,200	20.85	4.14	8	32	−0.04	−0.34
T3 loneliness	1,200	21.08	5.57	8	32	−0.01	−0.60
T1 depressive symptoms	1,200	13.92	4.48	0	27	−0.04	−0.60
T2 depressive symptoms	1,200	12.39	4.59	0	27	0.17	−0.42
T3 depressive symptoms	1,200	12.45	4.43	1	26	0.13	−0.34
T1 perceived social support	1,200	46.20	10.26	12	84	−0.01	−0.08

To improve transparency, the main text summarizes the key results of measurement invariance tests, the use of the four OSSS dimensions as indicators, the fixed item parceling principles for SIAS and ULS-8, the main specification of the revised CLPM, and the handling of the non-convergence of the RI-CLPM. Non-focal paths and technical outputs not presented in the main text were not interpreted as main results. The corresponding conclusions are based mainly on the focal paths, model fit, and sensitivity information reported in the main text.

Three supplementary analyses were further conducted. First, participants in the complete three-wave matched sample were compared with participants who were lost to follow-up or had incomplete data at later waves on T1 demographic variables and baseline psychological variables. Continuous variables were compared using Welch *t* tests with Cohen's d, and categorical variables were compared using χ^2^ tests with Cramer's *V*. Second, covariate coefficients from the fully adjusted observed-composite equations were reported. Third, the direction, magnitude, and significance of the focal longitudinal paths were compared across the minimally adjusted, demographic-adjusted, and fully adjusted models to examine whether the main conclusions depended on a specific covariate specification. Because AI-use purpose involved multiple categories and some subgroups were unevenly distributed, this study did not conduct full multi-group CLPMs by AI-use purpose and instead treated this issue as a direction for future research.

## 4 Results

### 4.1 Preliminary analyses and measurement invariance

Descriptive statistics are presented in Table 1. To ensure comparability of means and standard deviations across waves, Table 1 is based on the matched sample for the three-wave core variables (*N* = 1,200). Perceived support from AI chatbots was relatively stable across the three waves, and loneliness and social interaction anxiety also showed clear temporal stability. The main scales showed good internal consistency, with Cronbach's α values ranging from 0.84 to 0.94.

In this study, the PHQ-9 was used primarily as a control variable for general psychological distress and as a risk screening indicator. Based on the rechecked complete three-wave matched sample, using a PHQ-9 total score ≥ 10 as the screening threshold for moderate or higher depressive symptoms, the proportions meeting this threshold at T1, T2, and T3 were 69.5%, 55.0%, and 72.9%, respectively. This finding suggests a high level of depressive-symptom risk in the sample. Therefore, depressive symptoms were controlled in the main models, and all participants were provided with contact information for the school psychological counseling center and psychological crisis support resources as part of the ethical procedure. Because PHQ-9 item 9 concerns self-harm/suicidal ideation, it was not interpreted as a main study outcome and was used only for ethical risk prompting and help-seeking reminders at the end of the questionnaire.

Participants who reported higher-frequency risk responses on PHQ-9 item 9 additionally received a psychological crisis help-seeking prompt and referral information.

Longitudinal measurement invariance tests showed that configural, weak, and strong invariance models for OSSS, SIAS, and ULS-8 at the subscale or item-parcel level all reached acceptable levels, and ΔCFI and ΔRMSEA between adjacent models were within commonly used criteria. It should be noted that some CFI and TLI values in Supplementary Table S1 were close to or equal to 1, which may be related to the use of subscale/item-parcel indicators, low model degrees of freedom, and indicator aggregation. These values do not indicate “perfect” scale structure. Therefore, the results mainly support the basic appropriateness of comparing total scores across three waves and do not replace full item-level longitudinal latent measurement models.

### 4.2 Attrition bias, covariate coefficients, and sensitivity analyses

The complete matched sample for the three-wave core variables included 1,200 participants, and 300 participants had valid T1 data but were lost to follow-up or had incomplete data at later waves. Comparisons showed no significant differences between the complete sample and the attrited/incomplete sample in gender, age, grade, major category, residence status, T1 perceived support from AI chatbots, T1 social interaction anxiety, T1 loneliness, T1 depressive symptoms, or T1 real-life perceived social support. The largest absolute effect size for continuous variables was |d| = 0.125, and the largest Cramer's *V* for categorical variables was 0.050, both indicating very small effects. Thus, the current data did not show clear evidence of substantial attrition bias.

In addition, this study reported covariate coefficients from the fully adjusted observed-composite equations and conducted sensitivity analyses of the focal paths. The sensitivity analyses compared three models: a minimally adjusted model, a demographic-adjusted model, and a fully adjusted model. As shown in Table 2, the direction and main interpretation of the focal paths remained consistent across model specifications: the T2 OSSS → T3 loneliness path remained negative, the OSSS → subsequent social interaction anxiety paths remained positive, and the social interaction anxiety → subsequent loneliness paths remained positive. The T1 OSSS → T2 loneliness path was weak and non-significant across models. These findings indicate that the main conclusions regarding compensatory associations and social-interaction-anxiety-related risk associations were not driven solely by covariate specification. Complete attrition bias tests, covariate coefficients, and full sensitivity analyses are provided in Supplementary Tables S2–S4.

**Table 2:** Summary of attrition bias and focal-path sensitivity analyses.

Focal path	Minimally adjusted model β, *p*	Demographic-adjusted model β, *p*	Fully adjusted model β, *p*
T1 OSSS → T2 loneliness	−0.029, *p* = 0.193	−0.030, *p* = 0.185	−0.042, *p* = 0.080
T2 OSSS → T3 loneliness	−0.091, *p* < 0.001	−0.089, *p* < 0.001	−0.165, *p* < 0.001
T1 OSSS → T2 social interaction anxiety	0.181, *p* < 0.001	0.180, *p* < 0.001	0.161, *p* < 0.001
T2 OSSS → T3 social interaction anxiety	0.185, *p* < 0.001	0.185, *p* < 0.001	0.154, *p* < 0.001
T1 social interaction anxiety → T2 loneliness	0.208, *p* < 0.001	0.208, *p* < 0.001	0.198, *p* < 0.001
T2 social interaction anxiety → T3 loneliness	0.283, *p* < 0.001	0.281, *p* < 0.001	0.231, *p* < 0.001

OSSS, perceived support from AI chatbots. The minimally adjusted model controlled for prior levels of the outcome variable and adjacent focal predictors. The demographic-adjusted model additionally controlled for gender, age, grade, major category, and residence status. The fully adjusted model further controlled for depressive symptoms, baseline real-life perceived social support, and relevant second-order autoregressive components. Values are standardized regression coefficients (β) and *p* values. Because class identifiers were not retained, standard errors and *p* values were not adjusted for potential class clustering and should be interpreted cautiously.

### 4.3 Cross-lagged panel model and longitudinal mediation

The initial observed-composite CLPM converged but did not fit the data sufficiently: χ^2^ (36) = 462.994, CFI = 0.957, TLI = 0.880, RMSEA = 0.089, 90% CI (0.082, 0.096), SRMR = 0.018. Although CFI and SRMR were acceptable, TLI < 0.90 and RMSEA > 0.08 suggested that the model still insufficiently reproduced the covariance matrix according to commonly used model fit criteria (Hu and Bentler, 1999). Because loneliness, social interaction anxiety, and perceived support from AI chatbots may all show cross-semester stability, adjacent-wave autoregressive paths alone may be insufficient to capture longer-term stability components. Therefore, without adding additional cross-construct long-lag paths, the revised model added T1 → T3 second-order autoregressive paths for the same constructs, including OSSS, social interaction anxiety, loneliness, and depressive symptoms, in addition to adjacent-wave autoregressive paths. Model fit improved: χ^2^ (32) = 317.369, CFI = 0.971, TLI = 0.910, RMSEA = 0.077, 90% CI (0.070, 0.085), SRMR = 0.014. Compared with the initial model, the nominal χ^2^ difference was Δχ^2^ (4) = 145.625, *p* < 0.001. Because a conventional χ^2^ difference test is not a strictly robust difference test under MLR estimation, this result is used mainly to illustrate substantive improvement in model fit. The model fit comparison is shown in Table 3. It should be clarified that this revision was made after the initial model showed inadequate fit and was guided by the theoretical rationale that the core variables may contain stability components across measurement intervals. Therefore, the revised CLPM should be understood as a better-fitting predictive model specification rather than a fully prespecified and theoretically confirmed causal model.

**Table 3:** Model fit comparison for the initial CLPM, revised CLPM, and RI-CLPM sensitivity test.

Model	Model specification	χ^2^ (df)	CFI	TLI	RMSEA (90% CI)	SRMR	Conclusion
Initial CLPM	Adjacent-wave autoregressive and cross-lagged paths	462.994 (36)	0.957	0.880	0.089 (0.082, 0.096)	0.018	TLI and RMSEA indicated inadequate fit
Revised CLPM	T1 → T3 second-order autoregressive paths for the same constructs added	317.369 (32)	0.971	0.910	0.077 (0.070, 0.085)	0.014	Fit improved; used as the main observed-composite model
RI-CLPM	Random-intercept cross-lagged model for core variables	No stable solution obtained	—	—	—	—	Did not converge stably; reported as a sensitivity analysis, with main conclusions based on the revised CLPM

Based on this model positioning, subsequent path coefficients are used to describe short-term longitudinal predictive associations after controlling for prior levels and covariates. Although the revised model fitted the data better than the initial model, model revision itself does not provide causal identification. The relevant conclusions require further testing in future research using preregistered model comparisons, additional measurement waves, and models that can distinguish stable between-person differences from within-person change.

In other words, adding T1 → T3 same-construct second-order autoregressive paths is theoretically reasonable because loneliness, social interaction anxiety, perceived support from AI chatbots, and depressive symptoms may all contain stability components spanning two measurement intervals. However, this revision also has a data-informed component because it was adopted after the initial model showed inadequate fit. Therefore, this study positions the revised CLPM as a short-term longitudinal predictive model that better reproduces the covariance structure, rather than as a preregistered, fully confirmatory causal model. This treatment helps prevent model-fit improvement from being misinterpreted as causal confirmation of the proposed theoretical mechanism.

The hypothesis testing results are summarized in Table 4. In the revised CLPM, H1 was partially supported. The cross-time prediction from T1 OSSS to T2 loneliness did not reach statistical significance (β = −0.043, *p* = 0.064), whereas T2 OSSS significantly negatively predicted T3 loneliness (β = −0.166, *p* < 0.001). This result indicates that the compensatory association between perceived support from AI chatbots and lower subsequent loneliness did not appear consistently across both adjacent time intervals. More cautiously, this study can only conclude that a significant compensatory longitudinal predictive association was observed from T2 to T3, but it cannot infer from this result that the T1 → T2 and T2 → T3 path strengths differed statistically.

**Table 4:** Hypothesis testing results.

Hypothesis	Path	Beta	*p*	Conclusion
H1	T1 OSSS → T2 ULS	−0.043	=0.064	Not significant; no inference can be made about differences in path strength between the two intervals
H1	T2 OSSS → T3 ULS	−0.166	< 0.001	Partially supported; short-term predictive association
H2	T1 OSSS → T2 SIAS	0.161	< 0.001	Supported
H2	T2 OSSS → T3 SIAS	0.158	< 0.001	Supported
H3	T1 SIAS → T2 ULS	0.198	< 0.001	Supported
H3	T2 SIAS → T3 ULS	0.232	< 0.001	Supported
H4	T1 OSSS → T2 SIAS → T3 ULS	0.038	95% CI (0.026, 0.050)	Supported; statistical indirect predictive association
H5	T1 MSPSS → T2 SIAS	−0.042	=0.121	Not supported
H5	T1 MSPSS → T2 ULS	−0.051	=0.046	Partially supported
H5	T1 MSPSS → T3 SIAS	−0.166	< 0.001	Partially supported
H5	T1 MSPSS → T3 ULS	−0.449	< 0.001	Partially supported
H6	T2 OSSS × T1 MSPSS → T3 SIAS	−0.078	< 0.001	Supported
H6	T2 OSSS × T1 MSPSS → T3 ULS	−0.132	< 0.001	Supported; the low-support group showed no significant compensatory benefit

H2, H3, and H4 were supported. T1 OSSS significantly positively predicted T2 social interaction anxiety (β = 0.161, *p* < 0.001), and T2 OSSS significantly positively predicted T3 social interaction anxiety (β = 0.158, *p* < 0.001), supporting H2. T1 social interaction anxiety significantly positively predicted T2 loneliness (β = 0.198, *p* < 0.001), and T2 social interaction anxiety significantly positively predicted T3 loneliness (β = 0.232, *p* < 0.001), supporting H3. The longitudinal mediation analysis showed that T1 OSSS indirectly predicted T3 loneliness through T2 social interaction anxiety, with a standardized indirect effect of 0.038 and a 95% bootstrap CI (0.026, 0.050), supporting H4. If T1/T2 social interaction anxiety also positively predicted OSSS at the following wave, this should be interpreted as indicating that students with higher social anxiety may also be more inclined to seek or perceive AI support. Therefore, the results should be understood as short-term longitudinal predictive associations after controlling for prior levels, rather than as causal effects of perceived support from AI chatbots on loneliness or social interaction anxiety.

To enhance effect-size interpretation, unstandardized coefficients were also reported. The revised CLPM showed that each one-point increase in raw T2 OSSS was associated with an expected 0.071-point decrease in T3 ULS-8 loneliness (*B* = −0.071, SE = 0.006). Based on the sample standard deviation of T2 OSSS (12.92), a one-standard-deviation increase in OSSS corresponded to an approximately 0.92-point decrease in raw T3 ULS-8 loneliness. Each one-point increase in T2 social interaction anxiety was associated with an expected 0.110-point increase in T3 loneliness (*B* = 0.110, SE = 0.007). Based on the standard deviation of T2 SIAS (11.83), this corresponded to an approximately 1.30-point increase in loneliness. These conversions help interpret effect size but do not imply clinically meaningful thresholds and should not be taken as evidence of intervention effects. The revised three-wave cross-lagged model is shown in Figure 2.

**Figure 2:**
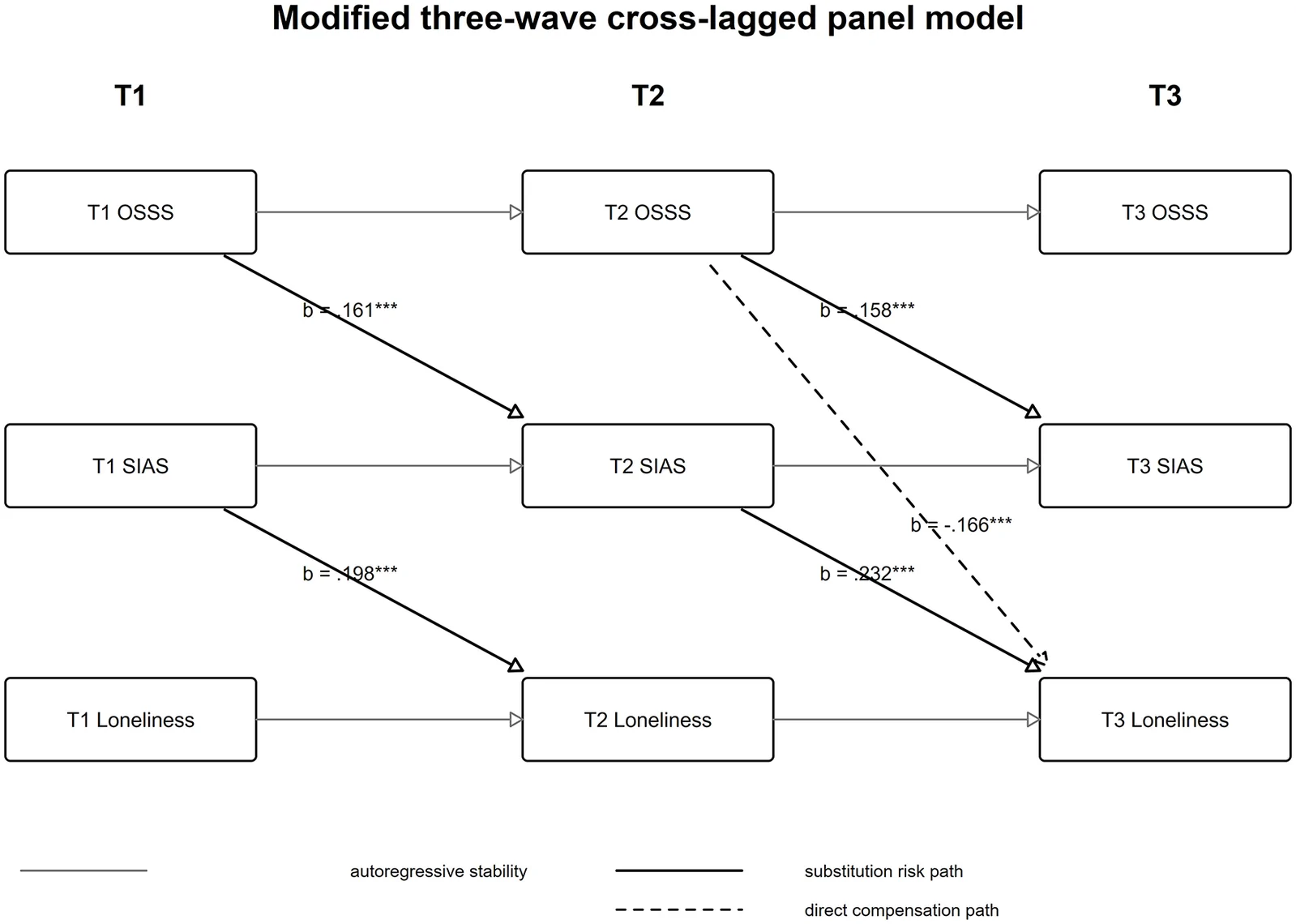
Revised three-wave cross-lagged panel model. Only focal paths are displayed. The full model also included autoregressive paths, within-wave residual correlations, T1 → T3 second-order autoregressive paths for the same constructs, and covariates. Only the main paths are shown. Covariates, within-wave residual correlations, and T1 to T3 second-order autoregressive paths are omitted for clarity.

### 4.4 Moderating role of real-life perceived social support

H5 and H6 were partially supported. For H5, T1 real-life perceived social support had a weak but significant negative predictive association with T2 loneliness (β = −0.051, *p* = 0.046), but its prediction of T2 social interaction anxiety did not reach significance (β = −0.042, *p* = 0.121). However, it showed significant protective predictive associations with both T3 social interaction anxiety (β = −0.166, *p* < 0.001) and T3 loneliness (β = −0.449, *p* < 0.001). Given the relatively large T1 MSPSS → T3 loneliness coefficient, this result should be understood as a strong longitudinal predictive association between baseline real-life perceived social support and later loneliness, rather than direct evidence of dynamic changes in real-life support. For H6, T1 real-life perceived social support significantly moderated the association between T2 OSSS and T3 social interaction anxiety (β = −0.078, *p* < 0.001) and also significantly moderated the association between T2 OSSS and T3 loneliness (β = −0.132, *p* < 0.001). Simple slope analyses showed that under high real-life perceived social support, T2 OSSS significantly negatively predicted T3 loneliness (slope = −0.287, *p* < 0.001); under low real-life perceived social support, this prediction was not significant (slope = −0.023, *p* > 0.05). This indicates that the low-support group was not harmed by AI support, but rather did not show a significant compensatory benefit. The conditional effect of T2 OSSS on T3 loneliness is shown in Figure 3.

**Figure 3:**
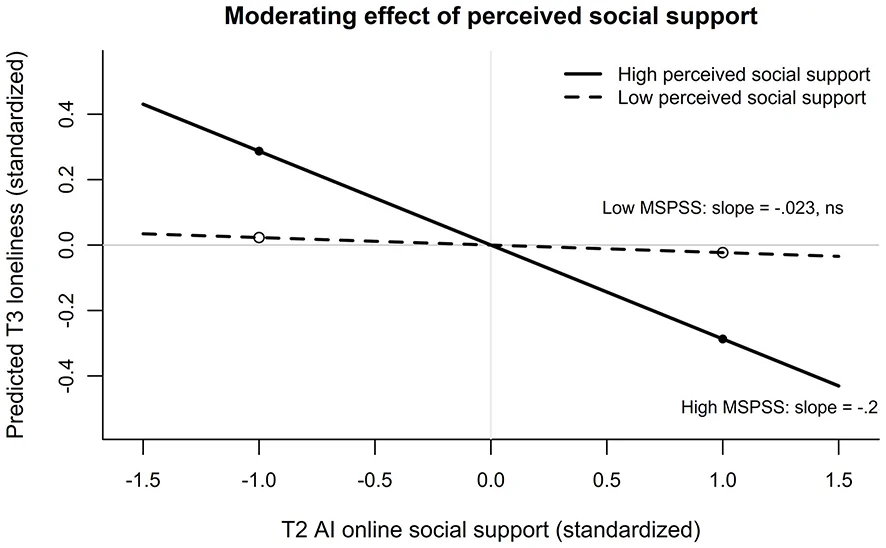
Moderating effect of T1 real-life perceived social support on the association between T2 OSSS and T3 loneliness. High and low real-life perceived social support represent one standard deviation above and below the mean, respectively. The y-axis represents predicted T3 loneliness in standardized units.

## 5 Discussion

This study aimed to answer a central question: whether perceived support from AI chatbots is closer to a compensatory resource in the development of loneliness among higher vocational college students, or whether it may form a risk-related longitudinal association through increases in subsequent social interaction anxiety. The revised CLPM results showed that the psychological meaning of perceived support from AI chatbots was not unidimensionally protective or risky, but conditional. On the one hand, T2 OSSS significantly predicted lower T3 loneliness, suggesting that AI support may have compensatory significance under certain conditions. On the other hand, OSSS significantly predicted subsequent social interaction anxiety across both adjacent intervals, and social interaction anxiety further predicted subsequent loneliness, indicating that AI support may also be linked to loneliness risk through a social-anxiety pathway. Because this study was a self-report, short-term, three-wave observational study and the RI-CLPM did not converge, the following discussion should be understood as an interpretation of longitudinal predictive associations rather than proof of causal mechanisms.

### 5.1 Why the dual-pathway results emerged

The compensatory pathway did not appear consistently across both adjacent intervals. One possible reason is that transforming AI chatbot responses from “immediate supportive experiences” into “psychological resources that can reduce loneliness” may require specific modes of use and contextual uptake. T1 OSSS did not significantly predict T2 loneliness, whereas T2 OSSS significantly predicted lower T3 loneliness, suggesting that supportive experiences initially obtained from AI may not immediately translate into reduced loneliness. This result is consistent with research viewing AI companionship as an accessible support resource (Jiao et al., 2026; Li et al., 2026), but it also differs from views that understand AI companionship as a direct tool for reducing loneliness, because this study shows that compensatory associations are constrained by time period and real-life support conditions. Another possible explanation is that T2–T3 corresponded to the later part of the semester, when academic pressure, peer relationships, and help-seeking needs may have become more concentrated, making AI responses more likely to be used for problem clarification, emotional organization, or help-seeking preparation. This explanation remains *post hoc* and should be tested in future research by directly measuring AI-use purposes and real-life help-seeking behavior.

Compared with the compensatory pathway, the social-interaction-anxiety-related risk association was more continuous. OSSS positively predicted social interaction anxiety at the following wave from T1 to T2 and from T2 to T3, and social interaction anxiety in turn continuously predicted loneliness at the following wave. Why might this pattern emerge? One possible reason is that AI interaction is controllable, immediate, and low in evaluative pressure. It may temporarily reduce expressive pressure but may not provide reciprocal feedback, conflict negotiation, or relationship repair opportunities that occur in real interpersonal interaction. This is consistent with risks suggested by research on problematic ChatGPT use and the boundaries of AI-based psychological support (Liao et al., 2026; Maral et al., 2025), but this study did not prove that AI support replaced real interaction. A more cautious interpretation is that higher perceived AI support was associated with higher subsequent social interaction anxiety, which may reflect that socially anxious students rely more on low-pressure interaction environments or that AI interaction may reduce opportunities for real social practice.

To avoid overinterpretation, this study describes this finding as a “social-interaction-anxiety-related risk association” rather than as a stronger behavioral substitution conclusion. This wording emphasizes that the study directly observed cross-time predictive associations among OSSS, social interaction anxiety, and loneliness, not a direct effect of AI use on reduced offline interaction or interpersonal substitution behavior. Future research can test the substitution interpretation only after including behavioral indicators such as offline interaction frequency, real help-seeking behavior, and the extent of AI substitution.

The findings on real-life perceived social support explain why AI support may have different meanings for different students. T1 real-life perceived social support did not significantly predict T2 social interaction anxiety, but it significantly predicted lower T3 social interaction anxiety and T3 loneliness. This may suggest that the protective effect of real-life support does not always appear immediately in the short term, but may emerge gradually through semester-long stress adjustment, peer relationship stabilization, and accumulation of help-seeking channels. This interpretation is consistent with the social support buffering model (Cohen and Wills, 1985) and with research on perceived social support, social anxiety, depressive symptoms, and loneliness among college students (Guo et al., 2026; Tang and Lu, 2026). Existing three-wave research also shows that perceived social support can buffer psychological risk among lonely individuals (Xu et al., 2020). However, because MSPSS was measured only at T1, this study cannot demonstrate changes in real-life support at T2 or T3 or test dynamic interactions between real-life support and AI support.

The moderation results for H6 further showed that the compensatory meaning of AI support requires real social support networks as a receiving condition. Under high real-life perceived social support, T2 OSSS significantly predicted lower T3 loneliness; under low real-life perceived social support, this association was not significant. This result is similar to the social enhancement perspective, which suggests that individuals with greater existing social resources are more likely to benefit from digital resources (Kraut et al., 2002; McKenna et al., 2002). Unlike traditional online social support, AI chatbots cannot directly provide reciprocal commitments in real relationships. Therefore, their compensatory role may depend more strongly on the uptake provided by real relationship networks. This study does not support the strong conclusion that “low-support students become lonelier because of AI support.” More precisely, when real-life support was insufficient, perceived support from AI chatbots did not significantly translate into reduced subsequent loneliness.

### 5.2 Differences from existing research, theoretical implications, and directions for future research

This study is both consistent with and meaningfully different from existing digital communication research. Existing social compensation perspectives suggest that online interaction may help individuals with insufficient social resources obtain additional support, whereas social enhancement perspectives emphasize that individuals with greater existing social resources are more likely to benefit from online resources (Kraut et al., 2002; McKenna et al., 2002). This study extends this logic to the context of generative AI and finds that perceived support from AI chatbots is not fully equivalent to traditional online interpersonal support. Traditional online social support usually comes from real people, whereas OSSS in this study essentially refers to supportive experiences perceived by students while using AI chat tools. Thus, the theoretical contribution of this study is to extend traditional digital communication theory to the context of generative AI support and to suggest that whether the support source is a real interpersonal agent may change the psychological meaning of social support.

This construct boundary also affects the theoretical interpretation of the study. Traditional online social support usually involves the intentions, reciprocity, and relational commitments of real others, whereas perceived support from AI chatbots more strongly reflects the responsiveness, companionship, and instrumental help experienced by users during AI interaction. Therefore, this study does not claim that AI chatbots can provide social support equivalent to human relationships. Instead, it examines perceived support from AI chatbots as an emerging, technology-mediated subjective support experience.

This difference also changes the questions that future research should ask. Future studies should not ask only whether AI can reduce loneliness. They should further distinguish three processes: first, whether AI responses help students organize emotions and prepare for help-seeking; second, whether AI interaction reduces or delays offline help-seeking and real interaction; and third, whether AI support is received, corrected, and extended within real social support networks. Only by directly measuring offline help-seeking frequency, peer interaction quality, interpersonal avoidance, the extent to which AI substitutes for real communication, and whether human help-seeking occurs after AI responses can future research determine whether the “social-interaction-anxiety-related risk association” truly reflects a behavioral substitution process.

The longitudinal mediation result for H4 further indicates that the association between perceived support from AI chatbots and loneliness is not a simple direct association, but may be linked through the psychological pathway of social interaction anxiety. This result is consistent with longitudinal evidence that social anxiety and loneliness are interrelated over time (Akkus and Peker, 2026) and with studies showing associations among social anxiety, loneliness, and problematic digital behavior among college students (Zhou and Feng, 2025; Fang et al., 2025). Unlike prior studies that mainly focused on social media or smartphone use, this study locates the risk association in the context of supportive AI interaction, suggesting that generative AI may change how students obtain support, express difficulties, and face real interpersonal situations.

This result also provides a contextual extension of the social support buffering model. The traditional social support buffering model emphasizes that social support can reduce the adverse impact of stress exposure on mental health (Cohen and Wills, 1985). This study further shows that, in the context of AI support, real-life perceived social support may not only directly predict lower subsequent loneliness and social interaction anxiety, but may also influence whether perceived support from AI chatbots shows a compensatory association. Future studies should repeatedly measure MSPSS at T1, T2, and T3 and compare the independent roles of family, friend, and significant-other support to test whether real-life support changes the psychological meaning of AI support over time.

These findings have three implications for future research. First, future studies should distinguish AI-use frequency, AI-use purpose, and perceived AI support, and examine different forms of use such as emotional venting, problem clarification, help-seeking preparation, companion dependence, academic assistance, and interpersonal substitution. Second, future research should use longer follow-up intervals and more waves, and should prioritize RI-CLPMs, latent growth models, or random-slope models to distinguish stable individual differences from within-person change. Third, future studies should combine self-reports, behavioral logs, platform-use records, and peer/counselor ratings to reduce the influence of common method bias, recall bias, and socially desirable responding.

### 5.3 Practical implications

In practical terms, the single-path effects observed in this study were small and should be understood as exploratory clues for population-level prevention. A one-standard-deviation increase in T2 OSSS corresponded to an approximately 0.92-point decrease in T3 ULS-8 loneliness, whereas a one-standard-deviation increase in T2 social interaction anxiety corresponded to an approximately 1.30-point increase in T3 loneliness. These effects suggest that colleges, when conducting AI literacy education and mental health education, may attend to how students use AI support and whether AI responses promote real help-seeking and interpersonal communication. Therefore, the following practical implications should be understood as design directions for future student-support programs and AI-literacy development, rather than as intervention programs already validated by the present study.

Accordingly, this study offers the following practical implications. First, at the student level, students may be guided to use AI for expression rehearsal, help-seeking script preparation, emotion recording, and problem clarification, rather than as a long-term substitute for peer communication and real help-seeking. Second, at the course and counseling levels, teachers, counselors, and mental-health staff may design learning or counseling points that encourage students to turn from AI responses to human support. For example, students may be encouraged to bring help-seeking outlines generated with AI into peer discussion, mentor communication, or psychological counseling. Third, at the platform and institutional levels, AI tools may include gentle reminders for real-life connection and referral prompts, especially when students repeatedly express loneliness, social fear, or self-harm risk, to guide them toward real interpersonal support and professional help. These suggestions do not come from an intervention experiment but are follow-up intervention-design hypotheses based on the longitudinal predictive associations observed in this study. In other words, the findings can provide risk-identification clues and mechanistic hypotheses for future support programs, but they cannot be used to infer the effectiveness of specific AI-literacy courses, psychological services, or platform reminder functions.

Thus, the practical significance of this study is not to demonstrate that a particular AI tool or course can already reduce loneliness. Rather, it is to propose testable design clues for future support programs: first, whether students use AI for help-seeking preparation rather than prolonged replacement of real communication; second, whether AI responses can be connected with peers, teachers, counselors, or psychological counseling resources; and third, whether AI-literacy education can include discussion of dependence, anthropomorphism, privacy, crisis referral, and maintenance of real relationships. These directions still require randomized controlled trials, quasi-experimental studies, or implementation research to evaluate their actual effects.

### 5.4 Limitations and future directions

This study has several limitations. First, although the revised CLPM substantially improved model fit, its RMSEA remained acceptable rather than excellent. This study attempted to estimate RI-CLPMs to distinguish stable between-person differences from within-person change, but neither the full version nor the simplified core-variable version achieved stable convergence. Possible reasons include high stability among core variables, a large number of model parameters, and limited information for random-intercept models in a three-wave design. Because the RI-CLPM did not converge, the main results should be interpreted as longitudinal predictive associations within the traditional CLPM framework rather than as strict within-person causal effects. This study used a short-term three-wave design within one semester with approximately 8-week adjacent intervals and therefore cannot explain cross-academic-year developmental trajectories or long-term causal processes. All variables were measured using self-report questionnaires and may be affected by common method bias, recall bias, and socially desirable responding. Although this study supplemented cross-wave invariance tests at the subscale/item-parcel level, full item-level longitudinal latent measurement invariance models have not yet been completed. In addition, perceived support from AI chatbots remains an emerging context-specific construct, and its discriminant validity from AI dependence, anthropomorphism, emotional attachment to AI, perceived responsiveness, and general AI-use intensity should be further tested in future studies using multiple methods, multiple samples, and full item-level measurement models. At the same time, adding T1 → T3 second-order autoregressive paths improved the model's reproduction of the data covariance structure, but the revised CLPM should still not be treated as a confirmed causal process model.

Second, MSPSS was measured only at T1. Therefore, this study could test only the boundary role of baseline real-life perceived social support and could not capture dynamic changes in real social support at T2 and T3.

Third, the sample came from four higher vocational colleges and was recruited through intact classes, but the original data did not retain class identifiers. Therefore, the number of participating classes at each institution and class-level sample sizes could not be reconstructed, and class-level ICCs, design effects, or cluster-robust/multilevel models could not be estimated. In addition, the number of institutions was only four, which was insufficient to support stable institution-level multilevel analysis. Thus, the results should be understood as individual-level longitudinal predictive associations. Future studies should retain anonymous class and institution codes while protecting participant privacy, to examine clustering structures and class/institution context effects. This study reported covariate coefficients, attrition bias tests, and focal-path sensitivity analyses that could be stably estimated. However, complete multi-group CLPMs by grade, major category, or AI-use purpose may still be affected by uneven subgroup sample sizes and model complexity. Future research should test these boundary conditions using larger samples and prespecified stratified sampling frameworks. Furthermore, because potential class clustering effects could not be estimated or adjusted, the standard errors, confidence intervals, and significance tests reported in this article may have been affected by non-independent observations. Statistical significance should therefore be interpreted cautiously together with effect sizes, consistency in path directions, and sensitivity-analysis results.

Fourth, this study did not directly measure real interpersonal interaction frequency, offline help-seeking, peer interaction quality, or the extent to which AI substituted for interpersonal interaction. Therefore, it cannot demonstrate whether AI support actually translated into real connection or replaced real relationships.

## 6 Conclusion

This study shows that perceived support from AI chatbots had both a compensatory longitudinal predictive association and a social-interaction-anxiety-related risk association with loneliness among higher vocational college students. The compensatory association was mainly reflected in T2 perceived support from AI chatbots predicting lower T3 loneliness. The social-interaction-anxiety-related risk association was reflected in perceived support from AI chatbots continuously predicting higher subsequent social interaction anxiety, which in turn predicted higher subsequent loneliness. T1 real-life perceived social support strengthened the compensatory association of AI support and weakened its risk association. Theoretically, this study extends the social compensation/social enhancement perspectives, the displacement hypothesis, and the social support buffering model from traditional digital communication to the context of generative AI support. Practically, the core implication is that colleges should guide students to use AI support tools for emotional organization, help-seeking preparation, and communication assistance, so that AI support can serve as a bridge to real social connection rather than as a substitute for real social connection. This implication is primarily intended for future AI-literacy education and student-support program design, and its actual effects still need to be tested in intervention research.

Accordingly, when college practitioners use these findings, they should treat them as a basis for identifying potential support needs and designing future service pathways, rather than treating AI chatbots themselves as validated loneliness intervention tools.

## Data Availability

The de-identified analytic data supporting the conclusions of this article will be made available by the corresponding author upon reasonable request. The data are not publicly deposited because participants' consent did not include permission for public sharing of raw data. Cross-wave matching codes and any information that could increase re-identification risk will not be shared.
