# Supplementary material for: Perceived support from AI chatbots and loneliness among higher vocational college students: a short-term three-wave study of social interaction anxiety and perceived social support
Source: Front Psychol. 2026 Sep 15;17:1928350. doi: 10.3389/fpsyg.2026.1928350 (PMC13619623; doi:10.3389/fpsyg.2026.1928350)
Supplement: Supplementary file 1 [file Supplementary_file_1.docx]

**Supplementary Material**

Dual longitudinal pathways linking artificial intelligence online social support to loneliness among higher vocational college students: a three-wave study of social interaction anxiety and perceived social support

# Supplementary Methods: measurement indicators and parceling

OSSS was repeatedly measured at T1, T2, and T3. For the longitudinal measurement invariance tests, the four theoretical OSSS domains were used as indicators at each wave: esteem/emotional support (items 1-5), social companionship (items 6-10), informational support (items 11-15), and instrumental support (items 16-20). Each domain score was computed as the arithmetic mean of its five items. The same indicator composition was retained across all three waves.

For SIAS, four item parcels were created at each wave using fixed item groupings: items 1-5, items 6-10, items 11-15, and items 16-20. For ULS-8, four item parcels were created at each wave using fixed item groupings: items 1-2, items 3-4, items 5-6, and items 7-8. Parcel scores were calculated as arithmetic means, and the same parceling procedure was applied at T1, T2, and T3. These parcel-level invariance tests were used to support the comparability of total scores across waves; they should not be interpreted as a substitute for full item-level longitudinal latent measurement models.

# Supplementary Table S1

Longitudinal measurement invariance tests for OSSS, SIAS, and ULS-8.

| **Scale** | **Model** | **chi-square** | **df** | **CFI** | **TLI** | **RMSEA** | **RMSEA 90% CI** | **SRMR** | **Delta CFI** | **Delta RMSEA** |
| --- | --- | --- | --- | --- | --- | --- | --- | --- | --- | --- |
| OSSS four-domain parcels | configural | 7.970 | 6 | 1.000 | 0.999 | 0.015 | [0.000, 0.033] | 0.002 |  |  |
| OSSS four-domain parcels | metric | 13.920 | 12 | 1.000 | 1.000 | 0.011 | [0.000, 0.027] | 0.007 | < .001 | -0.005 |
| OSSS four-domain parcels | scalar | 17.042 | 18 | 1.000 | 1.000 | 0.000 | [0.000, 0.020] | 0.007 | < .001 | -0.011 |
| SIAS item parcels | configural | 3.853 | 6 | 1.000 | 1.001 | 0.000 | [0.000, 0.012] | < .001 |  |  |
| SIAS item parcels | metric | 6.571 | 12 | 1.000 | 1.001 | 0.000 | [0.000, 0.000] | 0.003 | 0.000 | 0.000 |
| SIAS item parcels | scalar | 11.107 | 18 | 1.000 | 1.001 | 0.000 | [0.000, 0.000] | 0.004 | 0.000 | 0.000 |
| ULS-8 item parcels | configural | 8.396 | 6 | 1.000 | 0.999 | 0.018 | [0.000, 0.036] | 0.003 |  |  |
| ULS-8 item parcels | metric | 13.761 | 12 | 1.000 | 1.000 | 0.011 | [0.000, 0.029] | 0.008 | < .001 | -0.007 |
| ULS-8 item parcels | scalar | 23.207 | 18 | 0.999 | 0.999 | 0.015 | [0.000, 0.029] | 0.010 | < .001 | 0.004 |

Note. OSSS = AI online social support; SIAS = social interaction anxiety; ULS-8 = eight-item loneliness scale. Delta values are relative to the previous invariance model within the same scale.

# Supplementary Table S2

Complete revised CLPM paths, including non-significant and reciprocal cross-lagged paths.

| **Outcome** | **Predictor** | **Path type** | **B** | **SE** | **z** | **p** | **Beta (STDYX)** |
| --- | --- | --- | --- | --- | --- | --- | --- |
| T2 OSSS | T1 OSSS | Adjacent autoregressive path | 0.754 | 0.016 | 45.903 | 0.000 | 0.772 |
| T2 OSSS | T1 social interaction anxiety | Cross-lagged path | 0.067 | 0.018 | 3.689 | 0.000 | 0.067 |
| T2 OSSS | T1 loneliness | Cross-lagged path | -0.020 | 0.064 | -0.313 | 0.754 | -0.006 |
| T2 OSSS | T1 depressive symptoms | Cross-lagged path | -0.008 | 0.056 | -0.149 | 0.881 | -0.003 |
| T2 OSSS | T1 perceived social support | Covariate/control path | -0.013 | 0.024 | -0.552 | 0.581 | -0.010 |
| T2 OSSS | Gender | Covariate/control path | 0.289 | 0.413 | 0.700 | 0.484 | 0.011 |
| T2 OSSS | Age | Covariate/control path | -0.412 | 0.229 | -1.795 | 0.073 | -0.029 |
| T2 OSSS | Grade | Covariate/control path | 0.154 | 0.274 | 0.562 | 0.574 | 0.009 |
| T2 OSSS | Major | Covariate/control path | 0.155 | 0.186 | 0.836 | 0.403 | 0.013 |
| T2 social interaction anxiety | T1 social interaction anxiety | Adjacent autoregressive path | 0.440 | 0.025 | 17.361 | 0.000 | 0.483 |
| T2 social interaction anxiety | T1 OSSS | Cross-lagged path | 0.143 | 0.023 | 6.355 | 0.000 | 0.161 |
| T2 social interaction anxiety | T1 loneliness | Cross-lagged path | -0.030 | 0.084 | -0.354 | 0.723 | -0.010 |
| T2 social interaction anxiety | T1 depressive symptoms | Cross-lagged path | 0.142 | 0.074 | 1.907 | 0.057 | 0.054 |
| T2 social interaction anxiety | T1 perceived social support | Covariate/control path | -0.048 | 0.031 | -1.548 | 0.122 | -0.042 |
| T2 social interaction anxiety | Gender | Covariate/control path | 0.324 | 0.567 | 0.572 | 0.568 | 0.013 |
| T2 social interaction anxiety | Age | Covariate/control path | -0.365 | 0.300 | -1.216 | 0.224 | -0.028 |
| T2 social interaction anxiety | Grade | Covariate/control path | 0.251 | 0.368 | 0.681 | 0.496 | 0.016 |
| T2 social interaction anxiety | Major | Covariate/control path | -0.400 | 0.265 | -1.509 | 0.131 | -0.036 |
| T2 loneliness | T1 loneliness | Adjacent autoregressive path | 0.528 | 0.027 | 19.187 | 0.000 | 0.527 |
| T2 loneliness | T1 OSSS | Cross-lagged path | -0.013 | 0.007 | -1.847 | 0.065 | -0.043 |
| T2 loneliness | T1 social interaction anxiety | Cross-lagged path | 0.063 | 0.008 | 7.792 | 0.000 | 0.198 |
| T2 loneliness | T1 depressive symptoms | Cross-lagged path | 0.009 | 0.025 | 0.371 | 0.710 | 0.010 |
| T2 loneliness | T1 perceived social support | Covariate/control path | -0.020 | 0.010 | -2.000 | 0.046 | -0.051 |
| T2 loneliness | Gender | Covariate/control path | 0.312 | 0.184 | 1.695 | 0.090 | 0.037 |
| T2 loneliness | Age | Covariate/control path | 0.010 | 0.097 | 0.103 | 0.918 | 0.002 |
| T2 loneliness | Grade | Covariate/control path | 0.060 | 0.120 | 0.496 | 0.620 | 0.011 |
| T2 loneliness | Major | Covariate/control path | -0.117 | 0.083 | -1.412 | 0.158 | -0.031 |
| T2 depressive symptoms | T1 depressive symptoms | Adjacent autoregressive path | 0.513 | 0.031 | 16.799 | 0.000 | 0.501 |
| T2 depressive symptoms | T1 OSSS | Cross-lagged path | 0.018 | 0.009 | 2.011 | 0.044 | 0.052 |
| T2 depressive symptoms | T1 social interaction anxiety | Cross-lagged path | 0.019 | 0.010 | 1.973 | 0.048 | 0.054 |
| T2 depressive symptoms | T1 loneliness | Cross-lagged path | 0.055 | 0.033 | 1.670 | 0.095 | 0.050 |
| T2 depressive symptoms | T1 perceived social support | Covariate/control path | 0.000 | 0.012 | -0.029 | 0.977 | -0.001 |
| T2 depressive symptoms | Gender | Covariate/control path | -0.005 | 0.220 | -0.022 | 0.983 | -0.001 |
| T2 depressive symptoms | Age | Covariate/control path | -0.017 | 0.126 | -0.134 | 0.893 | -0.003 |
| T2 depressive symptoms | Grade | Covariate/control path | 0.007 | 0.143 | 0.049 | 0.961 | 0.001 |
| T2 depressive symptoms | Major | Covariate/control path | -0.061 | 0.103 | -0.594 | 0.553 | -0.014 |
| T3 OSSS | T2 OSSS | Adjacent autoregressive path | 0.692 | 0.029 | 23.687 | 0.000 | 0.706 |
| T3 OSSS | T2 social interaction anxiety | Cross-lagged path | 0.054 | 0.022 | 2.394 | 0.017 | 0.050 |
| T3 OSSS | T2 loneliness | Cross-lagged path | 0.016 | 0.062 | 0.250 | 0.803 | 0.005 |
| T3 OSSS | T2 depressive symptoms | Cross-lagged path | -0.046 | 0.055 | -0.831 | 0.406 | -0.017 |
| T3 OSSS | T1 perceived social support | Covariate/control path | -0.035 | 0.025 | -1.438 | 0.151 | -0.029 |
| T3 OSSS | Gender | Covariate/control path | 0.481 | 0.476 | 1.011 | 0.312 | 0.018 |
| T3 OSSS | Age | Covariate/control path | 0.150 | 0.257 | 0.582 | 0.560 | 0.011 |
| T3 OSSS | Grade | Covariate/control path | 0.530 | 0.311 | 1.707 | 0.088 | 0.032 |
| T3 OSSS | Major | Covariate/control path | -0.028 | 0.222 | -0.128 | 0.898 | -0.002 |
| T3 OSSS | T1 OSSS | Second-order autoregressive path | 0.045 | 0.032 | 1.397 | 0.163 | 0.047 |
| T3 social interaction anxiety | T2 social interaction anxiety | Adjacent autoregressive path | 0.717 | 0.018 | 39.312 | 0.000 | 0.652 |
| T3 social interaction anxiety | T2 OSSS | Cross-lagged path | 0.159 | 0.015 | 10.361 | 0.000 | 0.158 |
| T3 social interaction anxiety | T2 loneliness | Cross-lagged path | -0.011 | 0.051 | -0.213 | 0.831 | -0.003 |
| T3 social interaction anxiety | T2 depressive symptoms | Cross-lagged path | 0.105 | 0.045 | 2.310 | 0.021 | 0.037 |
| T3 social interaction anxiety | T1 perceived social support | Covariate/control path | -0.210 | 0.021 | -10.025 | 0.000 | -0.166 |
| T3 social interaction anxiety | Gender | Covariate/control path | -0.532 | 0.374 | -1.425 | 0.154 | -0.020 |
| T3 social interaction anxiety | Age | Covariate/control path | 0.106 | 0.189 | 0.559 | 0.576 | 0.007 |
| T3 social interaction anxiety | Grade | Covariate/control path | -0.241 | 0.241 | -0.998 | 0.318 | -0.014 |
| T3 social interaction anxiety | Major | Covariate/control path | 0.023 | 0.172 | 0.134 | 0.893 | 0.002 |
| T3 social interaction anxiety | T1 social interaction anxiety | Second-order autoregressive path | 0.128 | 0.018 | 6.967 | 0.000 | 0.127 |
| T3 loneliness | T2 loneliness | Adjacent autoregressive path | 0.610 | 0.023 | 26.651 | 0.000 | 0.451 |
| T3 loneliness | T2 OSSS | Cross-lagged path | -0.071 | 0.006 | -11.296 | 0.000 | -0.166 |
| T3 loneliness | T2 social interaction anxiety | Cross-lagged path | 0.110 | 0.007 | 16.258 | 0.000 | 0.232 |
| T3 loneliness | T2 depressive symptoms | Cross-lagged path | -0.004 | 0.018 | -0.238 | 0.812 | -0.003 |
| T3 loneliness | T1 perceived social support | Covariate/control path | -0.243 | 0.009 | -27.443 | 0.000 | -0.449 |
| T3 loneliness | Gender | Covariate/control path | -0.045 | 0.151 | -0.299 | 0.765 | -0.004 |
| T3 loneliness | Age | Covariate/control path | -0.117 | 0.079 | -1.471 | 0.141 | -0.019 |
| T3 loneliness | Grade | Covariate/control path | 0.031 | 0.095 | 0.322 | 0.747 | 0.004 |
| T3 loneliness | Major | Covariate/control path | 0.062 | 0.071 | 0.879 | 0.379 | 0.012 |
| T3 loneliness | T1 loneliness | Second-order autoregressive path | 0.147 | 0.023 | 6.407 | 0.000 | 0.108 |
| T3 depressive symptoms | T2 depressive symptoms | Adjacent autoregressive path | 0.554 | 0.017 | 33.209 | 0.000 | 0.573 |
| T3 depressive symptoms | T2 OSSS | Cross-lagged path | 0.013 | 0.005 | 2.522 | 0.012 | 0.037 |
| T3 depressive symptoms | T2 social interaction anxiety | Cross-lagged path | 0.055 | 0.006 | 9.316 | 0.000 | 0.148 |
| T3 depressive symptoms | T2 loneliness | Cross-lagged path | 0.287 | 0.018 | 16.182 | 0.000 | 0.268 |
| T3 depressive symptoms | T1 perceived social support | Covariate/control path | -0.020 | 0.007 | -2.816 | 0.005 | -0.046 |
| T3 depressive symptoms | Gender | Covariate/control path | -0.087 | 0.126 | -0.693 | 0.488 | -0.010 |
| T3 depressive symptoms | Age | Covariate/control path | 0.005 | 0.066 | 0.077 | 0.939 | 0.001 |
| T3 depressive symptoms | Grade | Covariate/control path | 0.072 | 0.082 | 0.877 | 0.380 | 0.012 |
| T3 depressive symptoms | Major | Covariate/control path | -0.033 | 0.059 | -0.559 | 0.576 | -0.008 |
| T3 depressive symptoms | T1 depressive symptoms | Second-order autoregressive path | 0.141 | 0.018 | 8.069 | 0.000 | 0.143 |

Note. B = unstandardized coefficient; Beta (STDYX) = standardized coefficient from Mplus. The revised CLPM included adjacent autoregressive paths, reciprocal cross-lagged paths, T1-to-T3 second-order autoregressive paths for the same constructs, within-wave residual correlations, and covariates.
