# Supplementary material for: Perceived support from AI chatbots and loneliness among higher vocational college students: a short-term three-wave study of social interaction anxiety and perceived social support
Source: Front Psychol. 2026 Sep 15;17:1928350. doi: 10.3389/fpsyg.2026.1928350 (PMC13619623; doi:10.3389/fpsyg.2026.1928350)
Supplement: Supplementary file 2 [file Supplementary_file_2.docx]

**Supplementary Tables for the Second-Round Revision**

Note. .Abbreviations: OSSS = perceived support from AI chatbots; SIAS = social interaction anxiety; ULS-8 = loneliness; MSPSS = perceived social support; PHQ-9 = depressive symptoms. Standardized variables are denoted by “standardized”.

# Supplementary Table S2. Attrition Bias Tests

| **Variable** | **Complete-sample n** | **Complete-sample M** | **Complete-sample SD** | **Attrited/incomplete n** | **Attrited/incomplete M** | **Attrited/incomplete SD** | **Test** | **Statistic** | **p** | **Effect size** | **Effect-size metric** |
| --- | --- | --- | --- | --- | --- | --- | --- | --- | --- | --- | --- |
| Age | 1200 | 19.007 | .919 | 300 | 19.027 | .946 | Welch t test | -.329 | = .742 | -.022 | Cohen d |
| T1 perceived support from AI chatbots (OSSS) | 1200 | 59.064 | 13.403 | 300 | 59.093 | 12.960 | Welch t test | -.035 | = .972 | -.002 | Cohen d |
| T1 social interaction anxiety (SIAS) | 1200 | 41.132 | 12.965 | 300 | 41.672 | 13.177 | Welch t test | -.638 | = .524 | -.042 | Cohen d |
| T1 loneliness (ULS-8) | 1200 | 20.854 | 4.178 | 300 | 21.145 | 3.905 | Welch t test | -1.139 | = .255 | -.071 | Cohen d |
| T1 depressive symptoms (PHQ-9) | 1200 | 13.915 | 4.483 | 300 | 14.474 | 4.480 | Welch t test | -1.934 | = .054 | -.125 | Cohen d |
| T1 perceived social support (MSPSS) | 1200 | 46.203 | 10.261 | 300 | 46.056 | 10.560 | Welch t test | .216 | = .829 | .014 | Cohen d |
| Gender | 1200 |  |  | 300 |  |  | Chi-square test | .035 | = .853 | .005 | Cramer's V |
| Grade | 1200 |  |  | 300 |  |  | Chi-square test | .398 | = .820 | .016 | Cramer's V |
| Major category | 1200 |  |  | 300 |  |  | Chi-square test | 3.740 | = .291 | .050 | Cramer's V |
| Residence status | 1200 |  |  | 300 |  |  | Chi-square test | 2.166 | = .141 | .038 | Cramer's V |

Note. The complete sample refers to participants with valid data on the three-wave core variables (n = 1,200). The attrited/incomplete sample refers to participants with valid T1 data but missing or incomplete data in later waves (n = 300). Continuous variables were compared using Welch t tests and Cohen’s d; categorical variables were compared using chi-square tests and Cramer’s V.

# Supplementary Table S3. Covariate Coefficients in the Fully Adjusted Observed-Composite Equations

| **Equation** | **Outcome** | **Covariate** | **Estimate** | **SE** | **t** | **p** | **n** | **R²** |
| --- | --- | --- | --- | --- | --- | --- | --- | --- |
| T2 OSSS equation | T2 OSSS total score (standardized) | T1 PHQ-9 total score (standardized) | .004 | .022 | .164 | = .870 | 1200 | .629 |
| T2 OSSS equation | T2 OSSS total score (standardized) | T1 MSPSS total score (standardized) | -.015 | .021 | -.708 | = .479 | 1200 | .629 |
| T2 OSSS equation | T2 OSSS total score (standardized) | Gender (dummy coded) | .038 | .036 | 1.048 | = .295 | 1200 | .629 |
| T2 OSSS equation | T2 OSSS total score (standardized) | Age (standardized) | -.022 | .018 | -1.238 | = .216 | 1200 | .629 |
| T2 OSSS equation | T2 OSSS total score (standardized) | Grade dummy 2 | -.008 | .042 | -.199 | = .842 | 1200 | .629 |
| T2 OSSS equation | T2 OSSS total score (standardized) | Grade dummy 3 | .002 | .047 | .036 | = .971 | 1200 | .629 |
| T2 OSSS equation | T2 OSSS total score (standardized) | Major category dummy 2 | -.043 | .052 | -.820 | = .412 | 1200 | .629 |
| T2 OSSS equation | T2 OSSS total score (standardized) | Major category dummy 3 | .077 | .044 | 1.732 | = .083 | 1200 | .629 |
| T2 OSSS equation | T2 OSSS total score (standardized) | Major category dummy 4 | -.019 | .056 | -.342 | = .733 | 1200 | .629 |
| T2 OSSS equation | T2 OSSS total score (standardized) | Residence status (dummy coded) | -.065 | .050 | -1.299 | = .194 | 1200 | .629 |
| T2 SIAS equation | T2 SIAS total score (standardized) | T1 PHQ-9 total score (standardized) | .055 | .029 | 1.864 | = .063 | 1200 | .352 |
| T2 SIAS equation | T2 SIAS total score (standardized) | T1 MSPSS total score (standardized) | -.042 | .028 | -1.533 | = .126 | 1200 | .352 |
| T2 SIAS equation | T2 SIAS total score (standardized) | Gender (dummy coded) | .026 | .048 | .546 | = .585 | 1200 | .352 |
| T2 SIAS equation | T2 SIAS total score (standardized) | Age (standardized) | -.030 | .023 | -1.263 | = .207 | 1200 | .352 |
| T2 SIAS equation | T2 SIAS total score (standardized) | Grade dummy 2 | -.009 | .055 | -.165 | = .869 | 1200 | .352 |
| T2 SIAS equation | T2 SIAS total score (standardized) | Grade dummy 3 | .047 | .062 | .767 | = .443 | 1200 | .352 |
| T2 SIAS equation | T2 SIAS total score (standardized) | Major category dummy 2 | -.057 | .069 | -.827 | = .409 | 1200 | .352 |
| T2 SIAS equation | T2 SIAS total score (standardized) | Major category dummy 3 | -.091 | .059 | -1.546 | = .122 | 1200 | .352 |
| T2 SIAS equation | T2 SIAS total score (standardized) | Major category dummy 4 | -.089 | .074 | -1.200 | = .230 | 1200 | .352 |
| T2 SIAS equation | T2 SIAS total score (standardized) | Residence status (dummy coded) | -.101 | .067 | -1.519 | = .129 | 1200 | .352 |
| T2 loneliness equation | T2 ULS-8 total score (standardized) | T1 PHQ-9 total score (standardized) | .008 | .027 | .308 | = .758 | 1200 | .443 |
| T2 loneliness equation | T2 ULS-8 total score (standardized) | T1 MSPSS total score (standardized) | -.050 | .026 | -1.943 | = .052 | 1200 | .443 |
| T2 loneliness equation | T2 ULS-8 total score (standardized) | Gender (dummy coded) | .078 | .045 | 1.738 | = .083 | 1200 | .443 |
| T2 loneliness equation | T2 ULS-8 total score (standardized) | Age (standardized) | .001 | .022 | .056 | = .956 | 1200 | .443 |
| T2 loneliness equation | T2 ULS-8 total score (standardized) | Grade dummy 2 | .057 | .051 | 1.120 | = .263 | 1200 | .443 |
| T2 loneliness equation | T2 ULS-8 total score (standardized) | Grade dummy 3 | .027 | .057 | .480 | = .631 | 1200 | .443 |
| T2 loneliness equation | T2 ULS-8 total score (standardized) | Major category dummy 2 | -.059 | .064 | -.930 | = .353 | 1200 | .443 |
| T2 loneliness equation | T2 ULS-8 total score (standardized) | Major category dummy 3 | -.107 | .054 | -1.975 | = .049 | 1200 | .443 |
| T2 loneliness equation | T2 ULS-8 total score (standardized) | Major category dummy 4 | -.046 | .068 | -.677 | = .499 | 1200 | .443 |
| T2 loneliness equation | T2 ULS-8 total score (standardized) | Residence status (dummy coded) | .046 | .062 | .750 | = .453 | 1200 | .443 |
| T2 PHQ-9 equation | T2 PHQ-9 total score (standardized) | T1 PHQ-9 total score (standardized) | .498 | .030 | 16.767 | < .001 | 1200 | .333 |
| T2 PHQ-9 equation | T2 PHQ-9 total score (standardized) | T1 MSPSS total score (standardized) | -.001 | .028 | -.030 | = .976 | 1200 | .333 |
| T2 PHQ-9 equation | T2 PHQ-9 total score (standardized) | Gender (dummy coded) | -.002 | .049 | -.041 | = .967 | 1200 | .333 |
| T2 PHQ-9 equation | T2 PHQ-9 total score (standardized) | Age (standardized) | -.005 | .024 | -.196 | = .845 | 1200 | .333 |
| T2 PHQ-9 equation | T2 PHQ-9 total score (standardized) | Grade dummy 2 | .006 | .056 | .105 | = .916 | 1200 | .333 |
| T2 PHQ-9 equation | T2 PHQ-9 total score (standardized) | Grade dummy 3 | .005 | .063 | .079 | = .937 | 1200 | .333 |
| T2 PHQ-9 equation | T2 PHQ-9 total score (standardized) | Major category dummy 2 | -.111 | .070 | -1.584 | = .113 | 1200 | .333 |
| T2 PHQ-9 equation | T2 PHQ-9 total score (standardized) | Major category dummy 3 | -.062 | .059 | -1.035 | = .301 | 1200 | .333 |
| T2 PHQ-9 equation | T2 PHQ-9 total score (standardized) | Major category dummy 4 | -.039 | .075 | -.521 | = .603 | 1200 | .333 |
| T2 PHQ-9 equation | T2 PHQ-9 total score (standardized) | Residence status (dummy coded) | .045 | .068 | .660 | = .510 | 1200 | .333 |
| T3 OSSS equation | T3 OSSS total score (standardized) | T2 PHQ-9 total score (standardized) | -.017 | .020 | -.857 | = .391 | 1200 | .587 |
| T3 OSSS equation | T3 OSSS total score (standardized) | T1 MSPSS total score (standardized) | -.027 | .021 | -1.280 | = .201 | 1200 | .587 |
| T3 OSSS equation | T3 OSSS total score (standardized) | Gender (dummy coded) | .038 | .039 | .975 | = .330 | 1200 | .587 |
| T3 OSSS equation | T3 OSSS total score (standardized) | Age (standardized) | .012 | .019 | .644 | = .520 | 1200 | .587 |
| T3 OSSS equation | T3 OSSS total score (standardized) | Grade dummy 2 | .030 | .044 | .687 | = .492 | 1200 | .587 |
| T3 OSSS equation | T3 OSSS total score (standardized) | Grade dummy 3 | .081 | .049 | 1.652 | = .099 | 1200 | .587 |
| T3 OSSS equation | T3 OSSS total score (standardized) | Major category dummy 2 | .025 | .055 | .447 | = .655 | 1200 | .587 |
| T3 OSSS equation | T3 OSSS total score (standardized) | Major category dummy 3 | .032 | .047 | .688 | = .492 | 1200 | .587 |
| T3 OSSS equation | T3 OSSS total score (standardized) | Major category dummy 4 | -.033 | .059 | -.558 | = .577 | 1200 | .587 |
| T3 OSSS equation | T3 OSSS total score (standardized) | Residence status (dummy coded) | .076 | .053 | 1.426 | = .154 | 1200 | .587 |
| T3 SIAS equation | T3 SIAS total score (standardized) | T2 PHQ-9 total score (standardized) | .033 | .015 | 2.220 | = .027 | 1200 | .769 |
| T3 SIAS equation | T3 SIAS total score (standardized) | T1 MSPSS total score (standardized) | -.162 | .016 | -10.265 | < .001 | 1200 | .769 |
| T3 SIAS equation | T3 SIAS total score (standardized) | Gender (dummy coded) | -.043 | .029 | -1.488 | = .137 | 1200 | .769 |
| T3 SIAS equation | T3 SIAS total score (standardized) | Age (standardized) | .006 | .014 | .440 | = .660 | 1200 | .769 |
| T3 SIAS equation | T3 SIAS total score (standardized) | Grade dummy 2 | -.058 | .033 | -1.742 | = .082 | 1200 | .769 |
| T3 SIAS equation | T3 SIAS total score (standardized) | Grade dummy 3 | -.033 | .037 | -.903 | = .367 | 1200 | .769 |
| T3 SIAS equation | T3 SIAS total score (standardized) | Major category dummy 2 | -.001 | .041 | -.024 | = .980 | 1200 | .769 |
| T3 SIAS equation | T3 SIAS total score (standardized) | Major category dummy 3 | -.008 | .035 | -.227 | = .821 | 1200 | .769 |
| T3 SIAS equation | T3 SIAS total score (standardized) | Major category dummy 4 | .007 | .044 | .150 | = .881 | 1200 | .769 |
| T3 SIAS equation | T3 SIAS total score (standardized) | Residence status (dummy coded) | -.093 | .040 | -2.333 | = .020 | 1200 | .769 |
| T3 loneliness equation | T3 ULS-8 total score (standardized) | T2 PHQ-9 total score (standardized) | -.003 | .014 | -.205 | = .837 | 1200 | .796 |
| T3 loneliness equation | T3 ULS-8 total score (standardized) | T1 MSPSS total score (standardized) | -.447 | .015 | -29.188 | < .001 | 1200 | .796 |
| T3 loneliness equation | T3 ULS-8 total score (standardized) | Gender (dummy coded) | -.009 | .027 | -.329 | = .742 | 1200 | .796 |
| T3 loneliness equation | T3 ULS-8 total score (standardized) | Age (standardized) | -.020 | .013 | -1.533 | = .126 | 1200 | .796 |
| T3 loneliness equation | T3 ULS-8 total score (standardized) | Grade dummy 2 | -.022 | .031 | -.699 | = .485 | 1200 | .796 |
| T3 loneliness equation | T3 ULS-8 total score (standardized) | Grade dummy 3 | .015 | .035 | .434 | = .665 | 1200 | .796 |
| T3 loneliness equation | T3 ULS-8 total score (standardized) | Major category dummy 2 | .045 | .039 | 1.173 | = .241 | 1200 | .796 |
| T3 loneliness equation | T3 ULS-8 total score (standardized) | Major category dummy 3 | .009 | .033 | .276 | = .782 | 1200 | .796 |
| T3 loneliness equation | T3 ULS-8 total score (standardized) | Major category dummy 4 | .053 | .041 | 1.268 | = .205 | 1200 | .796 |
| T3 loneliness equation | T3 ULS-8 total score (standardized) | Residence status (dummy coded) | -.060 | .037 | -1.592 | = .112 | 1200 | .796 |
| T3 PHQ-9 equation | T3 PHQ-9 total score (standardized) | T2 PHQ-9 total score (standardized) | .569 | .017 | 33.680 | < .001 | 1200 | .774 |
| T3 PHQ-9 equation | T3 PHQ-9 total score (standardized) | T1 MSPSS total score (standardized) | -.046 | .016 | -2.964 | = .003 | 1200 | .774 |
| T3 PHQ-9 equation | T3 PHQ-9 total score (standardized) | T1 PHQ-9 total score (standardized) | .151 | .018 | 8.296 | < .001 | 1200 | .774 |
| T3 PHQ-9 equation | T3 PHQ-9 total score (standardized) | Gender (dummy coded) | -.020 | .029 | -.691 | = .490 | 1200 | .774 |
| T3 PHQ-9 equation | T3 PHQ-9 total score (standardized) | Age (standardized) | -.000 | .014 | -.003 | = .998 | 1200 | .774 |
| T3 PHQ-9 equation | T3 PHQ-9 total score (standardized) | Grade dummy 2 | .012 | .033 | .352 | = .725 | 1200 | .774 |
| T3 PHQ-9 equation | T3 PHQ-9 total score (standardized) | Grade dummy 3 | .035 | .037 | .962 | = .336 | 1200 | .774 |
| T3 PHQ-9 equation | T3 PHQ-9 total score (standardized) | Major category dummy 2 | -.060 | .041 | -1.472 | = .141 | 1200 | .774 |
| T3 PHQ-9 equation | T3 PHQ-9 total score (standardized) | Major category dummy 3 | -.034 | .035 | -.968 | = .333 | 1200 | .774 |
| T3 PHQ-9 equation | T3 PHQ-9 total score (standardized) | Major category dummy 4 | -.021 | .044 | -.476 | = .634 | 1200 | .774 |
| T3 PHQ-9 equation | T3 PHQ-9 total score (standardized) | Residence status (dummy coded) | -.049 | .039 | -1.246 | = .213 | 1200 | .774 |

Note. The table reports covariate coefficients from the fully adjusted observed-composite equations. Categorical predictors were dummy coded according to the coding scheme used in the main analysis. Estimates are standardized when the outcome and continuous predictor were standardized.

# Supplementary Table S4. Sensitivity Analyses of the Focal Longitudinal Paths

| **Model** | **Path** | **β** | **SE** | **t** | **p** | **n** | **R²** |
| --- | --- | --- | --- | --- | --- | --- | --- |
| Minimally adjusted model | T1 OSSS → T2 loneliness | -.029 | .023 | -1.301 | = .193 | 1200 | .437 |
| Minimally adjusted model | T2 OSSS → T3 loneliness | -.091 | .018 | -5.076 | < .001 | 1200 | .647 |
| Minimally adjusted model | T1 OSSS → T2 social interaction anxiety | .181 | .024 | 7.447 | < .001 | 1200 | .345 |
| Minimally adjusted model | T2 OSSS → T3 social interaction anxiety | .185 | .015 | 12.019 | < .001 | 1200 | .744 |
| Minimally adjusted model | T1 social interaction anxiety → T2 loneliness | .208 | .025 | 8.479 | < .001 | 1200 | .437 |
| Minimally adjusted model | T2 social interaction anxiety → T3 loneliness | .283 | .019 | 15.164 | < .001 | 1200 | .647 |
| Demographic-adjusted model | T1 OSSS → T2 loneliness | -.030 | .023 | -1.327 | = .185 | 1200 | .441 |
| Demographic-adjusted model | T2 OSSS → T3 loneliness | -.089 | .018 | -4.923 | < .001 | 1200 | .649 |
| Demographic-adjusted model | T1 OSSS → T2 social interaction anxiety | .180 | .024 | 7.353 | < .001 | 1200 | .349 |
| Demographic-adjusted model | T2 OSSS → T3 social interaction anxiety | .185 | .015 | 11.969 | < .001 | 1200 | .747 |
| Demographic-adjusted model | T1 social interaction anxiety → T2 loneliness | .208 | .025 | 8.453 | < .001 | 1200 | .441 |
| Demographic-adjusted model | T2 social interaction anxiety → T3 loneliness | .281 | .019 | 15.000 | < .001 | 1200 | .649 |
| Fully adjusted model | T1 OSSS → T2 loneliness | -.042 | .024 | -1.753 | = .080 | 1200 | .443 |
| Fully adjusted model | T2 OSSS → T3 loneliness | -.165 | .014 | -11.697 | < .001 | 1200 | .796 |
| Fully adjusted model | T1 OSSS → T2 social interaction anxiety | .161 | .026 | 6.309 | < .001 | 1200 | .352 |
| Fully adjusted model | T2 OSSS → T3 social interaction anxiety | .154 | .015 | 10.216 | < .001 | 1200 | .769 |
| Fully adjusted model | T1 social interaction anxiety → T2 loneliness | .198 | .026 | 7.684 | < .001 | 1200 | .443 |
| Fully adjusted model | T2 social interaction anxiety → T3 loneliness | .231 | .014 | 15.920 | < .001 | 1200 | .796 |

Note. The minimally adjusted model controlled for the prior level of the outcome and adjacent focal predictors. The demographic-adjusted model additionally controlled for gender, age, grade, major category, and residence status. The fully adjusted model further controlled for depressive symptoms, baseline perceived social support, and relevant second-order autoregressive components.
